# Novel biochemical, structural, and systems insights into inflammatory signaling revealed by contextual interaction proteomics

**DOI:** 10.1073/pnas.2117175119

**Published:** 2022-09-30

**Authors:** Rodolfo Ciuffa, Federico Uliana, Jonathan Mannion, Martin Mehnert, Tencho Tenev, Cathy Marulli, Ari Satanowski, Lena Maria Leone Keller, Pilar Natalia Rodilla Ramírez, Alessandro Ori, Matthias Gstaiger, Pascal Meier, Ruedi Aebersold

**Affiliations:** ^a^Institute of Molecular Systems Biology, ETH Zurich, 8093 Zurich, Switzerland;; ^b^ The Breast Cancer Now Toby Robins Research Centre, The Institute of Cancer Research, SW3 6JB London, United Kingdom;; ^c^Max Planck Institute of Molecular Plant Physiology, 14476 Potsdam, Germany;; ^d^Institute of Molecular Biology and Biophysics, ETH Zurich, 8093 Zurich, Switzerland;; ^e^Leibniz Institute on Aging, Fritz Lipmann Institute, 07745 Jena, Germany;; ^f^Faculty of Science, University of Zurich, 8093 Zurich, Switzerland

**Keywords:** contextual proteomics, interaction proteomics, stoichiometry, inflammatory signaling, TNF-RSC

## Abstract

Protein–protein interactions (PPIs) represent the main mode of the proteome organization in the cell. In the last decade, several large-scale representations of PPI networks have captured generic aspects of the functional organization of network components but mostly lack the context of cellular states. However, the generation of context-dependent PPI networks is essential for structural and systems-level modeling of biological processes—a goal that remains an unsolved challenge. Here we describe an experimental/computational strategy to achieve a modeling of PPIs that considers contextual information. This strategy defines the composition, stoichiometry, temporal organization, and cellular requirements for the formation of target assemblies. We used this approach to generate an integrated model of the formation principles and architecture of a large signalosome, the TNF–receptor signaling complex (TNF-RSC). Overall, we show that the integration of systems- and structure-level information provides a generic, largely unexplored link between the modular proteome and cellular function.

## Significance

In this work, we propose a shift in the way we analyze and conceptualize protein–protein interaction (PPI) networks. We present an experimental and computational framework to model them in the cellular context. We applied this framework to the signalosome tumor necrosis factor–receptor signaling complex (TNF-RSC) and generated an integrated model of its formation and architecture that provides insights and resolved controversies regarding its organization and regulation. To achieve this, we first developed and optimized approaches to map the composition of a target complex, its absolute stoichiometry, its assembly dynamics, its temporal dependence on signaling, and its reliance on the expressed proteome.

As a consequence of the emergence of disruptive technologies, most classes of biomolecules can now be systematically measured. Typically, such omic technologies are initially used to generate generic, decontextualized maps of the respective molecular class, followed by subsequent studies that aim at relating the respective “ome” to the context of specific cell types or cellular states. This trajectory, exemplified by advances in genomics, transcriptomics, or proteomics, has proven invaluable for basic and translational research.

The application of omic technologies to the study of protein–protein interactions (PPIs) has shown that protein assemblies mediate most biological functions (1). Such assemblies, whether stable or transient, can undergo dramatic changes in their composition, topology, subcellular localization, and activity as a function of the cellular state. To date, substantial progress has been made to generate generic maps of protein complexes and PPIs (2–4). These maps are essential to drive discoveries and define general properties of the proteome organization, such as the definition of hub proteins, but they typically lack the contextual information that relates specific assemblies to the availability of cellular resources and cellular functional state. In essence, the systematic exploration of the modularity of the proteome has yet to achieve the transition from deconceptualized maps to measurements in the context of cellular state. To bring about this transition, several layers of data are required: 1) an accurate map of the assembly composition; 2) information about its level of organization, such as size, subunit stoichiometry, and formation principles; 3) a description of its quantitative temporal alteration as function of the cell state (e.g., signaling activation); 4) its cellular coordinates and the resources that enable and constrain its formation; and 5) a conceptual/computational framework to integrate structure- and systems-level information.

**Fig. 1.**
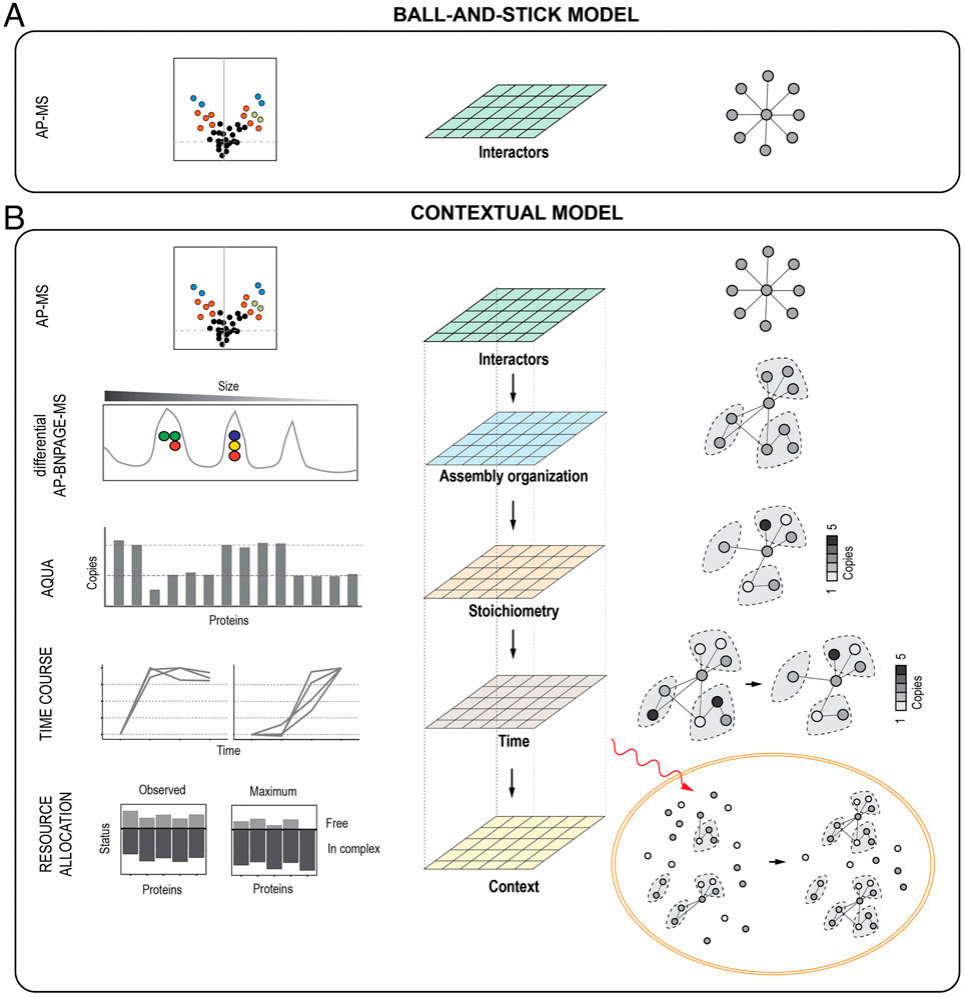
PPI contextual modeling approach. (*A*) In typical AP-MS workflow, PPI networks are generated from a single layer of data: those proteins that are identified as differentially abundant against a control (volcano plot; *Left*). (*B*) To achieve a contextual modeling, we built on the first interaction layer and further characterized a target assembly by a differential native separation by BNPAGE (AP-BNPAGE-MS), absolute quantification with AQUA grade peptides over time after stimulation, and determination of cellular resources distribution during signaling. The integration of these different layers can be used to describe constraints dictated by available resources and structural properties on the formation of assemblies and their activity.

In this study, we combined and further developed orthogonal mass spectrometric (MS) approaches to address these challenges. They include analyses of a target assembly by different MS modalities; a differential native separation method to study its disassembly; an in-depth, time-resolved determination of its absolute stoichiometry from affinity-purified samples(AP-absolute quantification [AQUA grade peptides]-MS); the combination of absolute quantification in lysates (lysate-AQUA) and affinity-purified samples to quantify the proportion of cytosolic proteins assembled in complex; and a framework to combine all these data layers in a model (Fig. 1). We selected as case study the tumor necrosis factor–receptor signaling complex (TNF-RSC), a signalosome that plays a pivotal role in both inflammation and cancer (5, 6). This system was selected for three reasons: 1) it represents a challenging example of low-abundance, membrane-bound signalosome that forms only transiently and in a posttranslational modification (PTM)-dependent fashion; 2) there is a large body of literature that can be used to both benchmark and complement our results; and 3) despite this, several important questions remain unaddressed regarding its regulation, structural organization, and systems-level properties. These include, for instance, regulation by phosphatases and architecture of the partaking protein complexes. In summary, we present a generic, widely applicable strategy for the contextual modeling of protein assemblies, and by applying it to a challenging membrane-bound immunological signalosome, we provide insights into its biochemical, structural, and systems-level organization.

## Results

### First Layer of the Contextual Model: Identification of Interactors Using AP-MS.

**Fig. 2.**
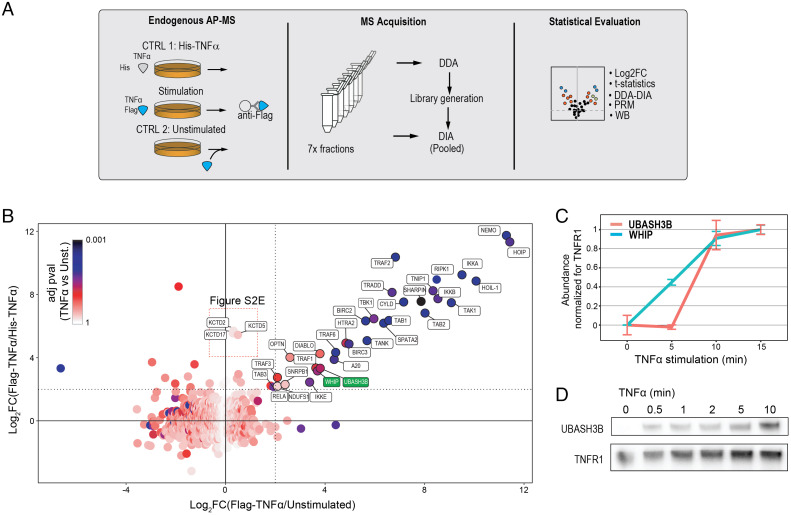
Landscape of TNF-RSC and characterization of UBASH3B and WHIP as a signalosome member. (*A*) Experiment design: A549 cells were stimulated and isolated using a Flag-tagged TNFα. Addition of Flag-tagged TNFα to unstimulated lysates or His-tagged TNFα to intact cells was used as control. Samples were fractionated and analyzed by DDA, while pooled ones were analyzed by DIA. Standard statistical procedures were used to determine high-confidence interactors, and additional analyses revealed midconfidence associated proteins. Finally, additional pulldowns and biochemical experiments were performed to validate UBASH3B and WHIP as TNF-RSC complex members. (*B*) Scatterplot showing protein enrichment across the two controls (His-tagged, *y* axes; unstimulated, *x* axes). Adjusted *P* value against the unstimulated control is coded in the dot color. (*C*) The recruitment of WHIP and UBASH3B to the TNF-RSC is confirmed by targeted proteomics on isolated signalosomes (A549 cells) across the indicated time points after stimulation. The data are based on the same experiment presented in *SI Appendix*, Fig. S13*A*. (*D*) Immunoblot analysis for the recruitment of UBASH3B to the TNF-RSC.

The composition of the TNF-RSC has been previously studied by MS following its isolation via tagged-TNFα (7, 8). In the first step of our workflow, we set out to map the TNF-RSC composition with high quantitative accuracy and confidence. This was achieved by using a fractionation step and two orthogonal controls and by adopting an analysis strategy based on multiple MS modalities (Fig. 2*A*) (*Materials and Methods*). We first analyzed, by data-dependent analysis (DDA)-MS, TNF-RSC isolated from A549 cells stimulated for 10 min with the cytokine TNFα (Fig. 2*B* and *SI Appendix*, Fig. S1 *A*, *B*, *D–F* and Datasets S1 and S2). As shown in the scatterplot in Fig. 2*B*, our results identify about 30 high-confidence interactors. To evaluate the performance of our filtering strategy, we constructed receiver operating characteristic curves for both controls. We used as a benchmark those proteins that have been reported in the protein-protein interactions repository BioGRID (v.4.4.205) (9) to physically associate with the receptor TNFR1 by at least three studies. As shown in *SI Appendix*, Fig. S2*A*, both controls achieve remarkable performance, with areas under the curve of 0.81 and 0.91, with the control relying on receptor stimulation with a His-tagged version of TNFα being highly selective and specific. Remarkably, we identified two new high-confidence interactors (the phosphatase UBASH3B and the triple ATPase WHIP; Fig. 2*B* and *SI Appendix*, Fig. S2*B*) and several less well characterized TNF-RSC–associated proteins at a lower level of confidence. These include AZI2 (a TBK1-interacting protein), TAX1BP1 (negatively regulating TNF-induced cell death) (*SI Appendix*, Fig. S2 *C* and *D*), and KCTD2/5/17 (a putative Cul3 subunit), which are enriched in a stimulus-independent fashion (Fig. 2 *B*, *Inset*, and *SI Appendix*, Fig. S2*E* and *Mid-confidence interactors identifications*, for a more extensive discussion). One of the two new high-confidence interactors, UBASH3B, is a phosphatase known to regulate EGFR receptor internalization and TCR signaling (10). The other member, WHIP, has been recently characterized as part of a trimeric complex involved in innate antiviral response (11). Furthermore, it also has been reported to interact with the ubiquitin ligase HOIP (together with SHARPIN and HOIL-1 part of the LUBAC complex) in a previous MS screen but, to our knowledge, never been associated with the TNF-RSC (12). Previous work established that WHIP contains a PUB interacting motif (PIM) with which it can bind to the PUB (PNGase/UBA or UBX-containing protein) domain of HOIP under in vitro settings (12).

To corroborate these interactions, we first analyzed by data-independent acquisition (DIA) the same samples (after pooling the fractions) used for the DDA analysis. Using this orthogonal data acquisition method, we could confirm the enrichment of both proteins (*SI Appendix*, Figs. S1 *C* and *G–I* and S2 *F* and *G* and Dataset S3). Next, we performed a time course analysis to evaluate the recruitment of UBASH3B and WHIP to the TNFR1 in response to TNFα stimulation (time points 0, 5, 10, and 15 min). To measure the association dynamics, we used parallel reaction monitoring (PRM), a MS data acquisition strategy that targets few analytes and quantifies them with high quantitative accuracy. As shown in Fig. 2*C*, our data indicate that both proteins associate with TNFR1 in a stimulus- and time-dependent manner. Based on PRM profiles, they seem to exhibit somewhat different recruitment dynamics, with WHIP being recruited already at 5 min while UBASH3B associates at 10 min. These data are confirmed by the recruitment of UBASH3B to the TNF-RSC by immunoblot analysis (Fig. 2*D*).

**Fig. 3.**
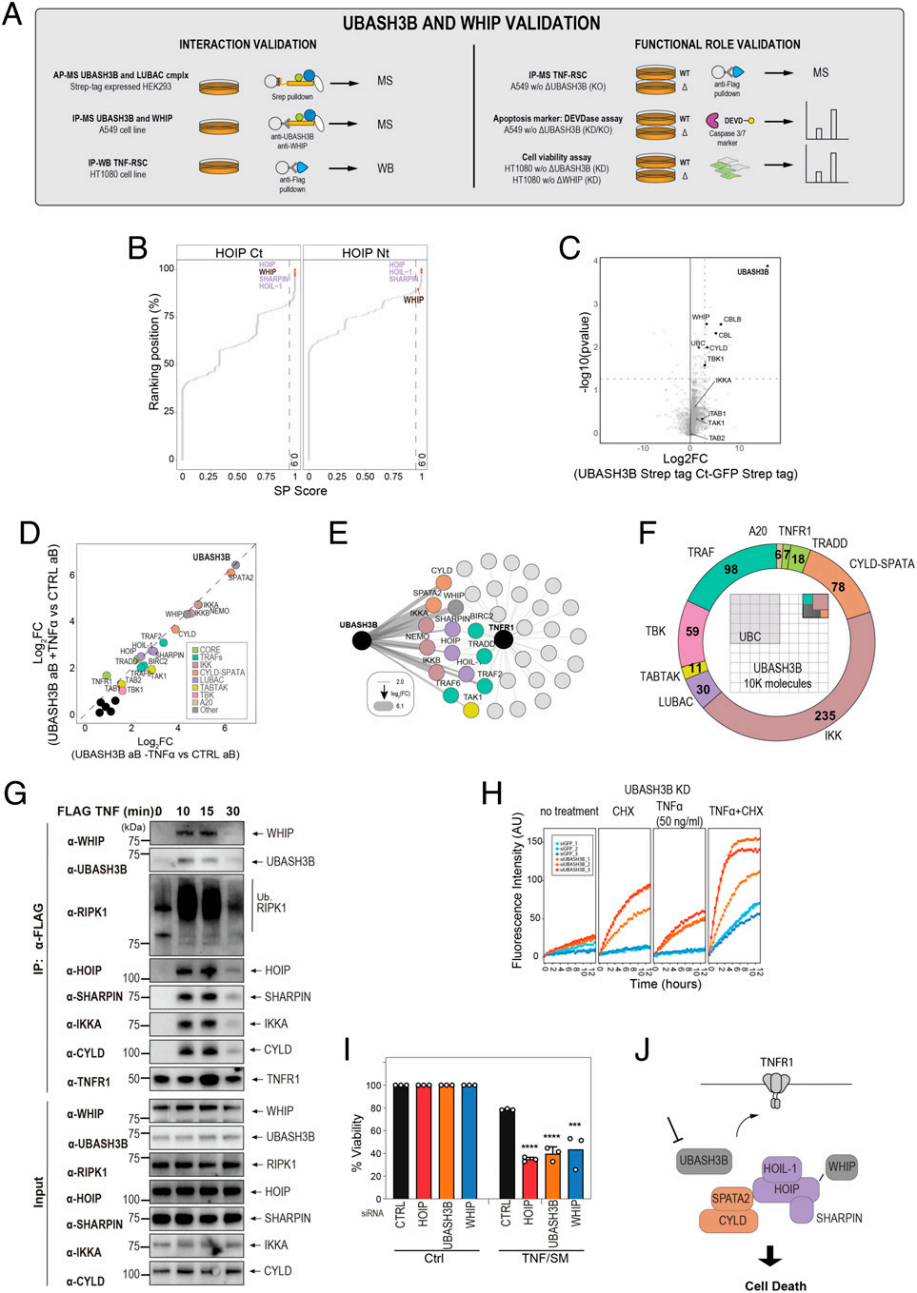
Functional and biochemical validation of UBASH3B and WHIP interaction with TNF-RSC. (*A*) Summary of validation experiments for UBASH3B and WHIP association with TNF-RSC. (*B*) Scatterplot of HOIP interactors identified by affinity purification of C- or N-terminally tagged HOIP. Saint score indicates the recruitment enrichment of interactors against three GFP controls. All identified known members and associated components of TNF-RSC are shown in the plot. LUBAC components and WHIP are highlighted in purple and black, respectively. (*C*) DDA-MS analysis of UBASH3B interactome. The volcano plot shows copurified proteins identified by affinity purification of C-terminally tagged UBASH3B ectopically expressed in HEK293 cells against a GFP control. (*D*) Targeted IP-MS analysis of endogenous UBASH3B in A549 cells. The scatterplot displays the enrichment of the untreated and TNFα-treated proteins isolated against a nonspecific serum control. (*E*) Network model showing overlap of proteins enriched with a log_2_FC threshold >2 in both UBASH3B IP-MS (on the left) and TNF-RSC affinity purification (on the right). Thickness of UBASH3B edges scales with enrichment of associated nodes. (*F*) Targeted IP-MS analysis of endogenous UBASH3B in A549 cells. Occupancy plot (inside) showing the fraction of the indicated interactors bound to an arbitrary number of UBASH3B molecules. The numbers are reported in the circular doughnut chart. (*G*) Affinity purification of TNF-RSC from HT1080 cells demonstrating that UBASH3B and WHIP are selectively copurified in a ligand and time-dependent manner. (*H*) DEVDase assay with the indicated siRNA (B10 from *SI Appendix*, Dataset S13) against UBASH3B. siGFP was used as a control. Profile of each replicate is shown. (*I*) UBASH3B and WHIP knock-down sensitizes HT1080 cells to TNF-induced cell death to the same extent as knock-down of HOIP. The indicated targets were knocked down by RNAi in HT1080 cells and cells treated with either DMSO control or TNF/SM. (*J*) Model of the recruitment and signaling role for UBASH3B and WHIP.

In the next step, we focused on characterizing the interaction between UBASH3B, WHIP, and TNF-RSC in orthogonal model systems, and we evaluated the functional role of this interaction in inflammation and apoptosis (Fig. 3*A*). To test whether WHIP interacts with HOIP, we performed AP-MS experiments using N- and/or C-terminally tagged SHARPIN, HOIL-1, and HOIP. The Saint algorithm (13) was used to identify proteins significantly associated with these baits. Consistent with Schaefer et al. (12), we found that endogenous WHIP was significantly enriched in HOIP isolates (for both N- and C-terminal tags; Fig. 3*B* and *SI Appendix*, Fig. S2 *H* and *I*; Saint score > 0.90, BFDR [Bayesian false discovery rate] < 0.05; Dataset S4). In contrast, the enrichment was not significant when SHARPIN or HOIL-1 were used as affinity reagents (*SI Appendix*, Fig. S2 *H* and *I*). Together, these data support the notion that WHIP is recruited to the TNF-RSC via its interaction with HOIP.

We next focused on UBASH3B. We evaluated the interaction between UBASH3B and components of the TNF-RSC in HEK293 cell lines: we isolated C- and N-terminally Strep-tagged UBASH3B and quantified the coisolated proteins by DDA-MS. Results are illustrated in the volcano plots in Fig. 3*C* (C-terminal AP-MS) and *SI Appendix*, Fig. S3 *A–E* (Dataset S4). With both tagged versions of UBASH3B, we found members of the TNF-RSC enriched several folds over the control, even though not significantly in the experiments carried out with the N-terminally tagged UBASH3B. Of note, and in line with the experiments carried out in A549 cells, CYLD and WHIP were consistently enriched. Next, we asked whether UBASH3B is interacting with TNF-RSC proteins also in steady-state and what TNF-RSC proteins it does most strongly interact with. To this end, we affinity purified endogenous UBASH3B from A549 cells using UBASH3B antibodies that were raised against its N terminus (Fig. 3 *D–F*; *SI Appendix*, Fig. S4 *A–G*; and Dataset S5). Using targeted MS acquisition strategy (PRM), we evaluated the presence of components of the TNF-RSC. Our data indicate that CYLD-SPATA2, the IKK complex members, and WHIP itself are significantly enriched in isolates of endogenous UBASH3B. Using synthetic peptides spiked into the sample to perform absolute quantification of endogenously expressed proteins, we found that the binding of TNF-RSC components to UBASH3B was, with the exception of ubiquitin, highly substoichiometric (Fig. 3*F*). To provide further evidence supporting the interaction of UBASH3B and WHIP with members of TNF-RSC, we performed affinity purification of TNF-RSC using a different model system (HT1080 fibroblast cell line) (Fig. 3*G*). In this experiment, UBASH3B and WHIP1 coimmunoprecipitated with complex I TNF-RSC with a dynamic following receptor stimulation that was comparable to that observed by PRM quantification (Fig. 2*C*).

Finally, we interrogated the function of UBASH3B and WHIP in the context of inflammatory signaling and apoptosis. To this aim, we first performed small interfering RNA (siRNA) interference experiment in A549 cells. We found that RNA interference (RNAi)-mediated down-regulation of UBASH3B led to an increase in TNFα-induced apoptosis, as measured by the DEVDase caspase activity assay by pooled (and one individual) siRNA (Fig. 3*H* and *SI Appendix*, Fig. S5 *A–C* and Dataset S6). Likewise, we found that knock-down of UBASH3B and WHIP in cells treated with TNFα and Smac mimetic SM164 reduced cell viability, with a phenotype comparable with the knock-down of other well-known components of the TNF-RSC (i.e., HOIP) (Fig. 3*I* and *SI Appendix*, Fig. S5*D*). Remarkably, the link between UBASH3B and TNF signaling is also supported by a recent large-scale phosphoproteomic screen (14), which indicates that phosphorylation of UBASH3B (S377) is consistently increased by TNF signaling (*SI Appendix*, Fig. S5*E*) and is negatively regulated by inhibition of the TNF-RSC member TAK1 and TNF-RSC effector p38 (*SI Appendix*, Fig. S5*F*). On the other hand, genetic ablation (knock-out [KO]) of UBASH3B in A549 cells does result in a mild proapoptotic response (*SI Appendix*, Fig. S5 *G–I*) and did not significantly perturb proinflammatory signaling, as measured by immunoblot analysis of phosphorylation of the downstream factors iκBα, p65, and MAP kinases (*SI Appendix*, Fig. S6). Furthermore, we found that the composition of the TNF-RSC affinity purified from A549 UBASH3B KO cells did not significantly differ from the wild type (WT), suggesting that UBASH3B is not required for the signalosome stabilization or the recruitment of any of its analyzed members (*SI Appendix*, Fig. S7 *A–E* and Dataset S7).

Taken together, these data point at a potential, nonessential role of UBASH3B in regulating the apoptotic response (Fig. 3*J*). Overall, by using orthogonal controls, MS approaches, and reciprocal affinity purifications, we could reliably detect the recruitment of known, and identify new components of the TNF-RSC signalosome.

### Second Layer of the Contextual Model: Investigation into Complex Assembly via AP-Blue Native Polyacrylamide Gel Electrophoresis-MS.

**Fig. 4.**
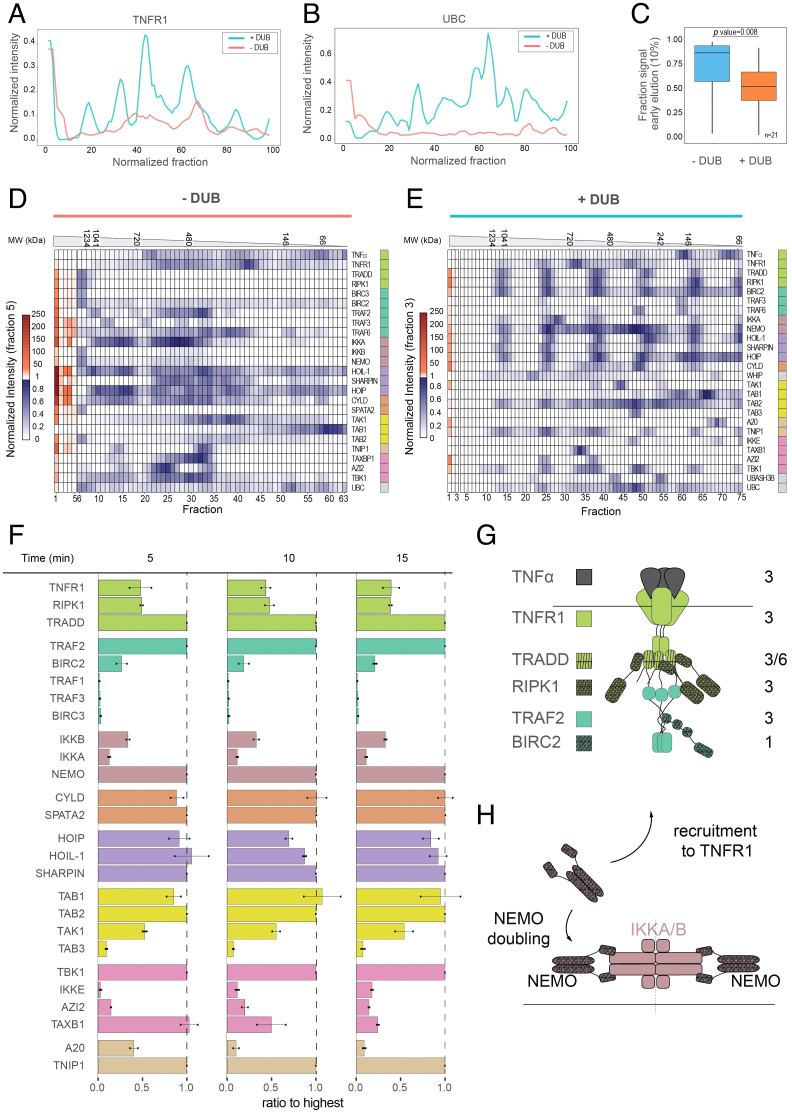
Architecture of the TNF-RSC. (*A* and *B*) Comparison of TNFR1 receptor and ubiquitin signal in the two conditions (±DUB) indicates increase in signal and peak sharpening in DUB-treated complexes. Signal is normalized to the maximum intensity. (*C*) Signal is significantly shifted from early to late fractions in DUB-treated samples, indicating complex disassembly. (*D*) Signal distribution for the untreated, BNPAGE-separated TNF-RSC proteins. Signal is normalized based on the intensity of fraction 5 (first relative minimum). (*E*) Signal distribution for the DUB-treated, BNPAGE-separated TNF-RSC proteins. Signal is normalized based on the intensity of fraction 3 (first relative minimum). (*F*) Bar plot of complex stoichiometries over time as determined by AP-AQUA-MS. (*G*) Model of the TNFR1 core complex based on AP-MS (iBAQ) and AP-AQUA-MS data approximates results from previous in vitro characterizations. (*H*) Model of the stoichiometry rearrangement of the IKK complex upon recruitment, with duplication of the NEMO subunit and the existence of distinct cellular isoforms of the IKKA/IKKB complex.

We next aimed at characterizing the organization of the TNF-RSC signalosome and the complexes that constitute it. To this aim, we first developed a targeted, differential blue native polyacrylamide gel electrophoresis (BNPAGE) approach to evaluate the size and modularity of the TNF-RSC signalosome (*SI Appendix*, Fig. S8*A*). This approach comprises five steps. First, we isolated endogenous TNF-RSC from a large amount of starting material (≥1 × 10^9^ cells), using the cytokine TNFα to both induce the formation and capture the signalosome, as shown in Fig. 2*A*. In a second step, one of two samples was treated with the USP21, a deubiquitylating enzyme that removes ubiquitin adducts irrespective of their linkage types (15) (*SI Appendix*, Fig. S8*B*). We reasoned that USP21-mediated abscission of ubiquitin helps to partially disassemble the TNF-RSC so that individual subcomplexes can be better resolved by nondenaturing BNPAGE. Third, we separated the digested (+DUB) or undigested (−DUB) isolated TNF-RSC by the nondenaturing BNPAGE (*SI Appendix*, Fig. S8 *F* and *G*). Fourth, to ensure high precision and quantification accuracy, we used a custom-built gel slicer to fractionate the BNPAGE in sixty-five to seventy-five 1-mm slices (*SI Appendix*, Fig. S8*C*). Finally, we analyzed the protein contents of consecutive slices via the sensitive, targeted PRM method, providing sensitivity in the zeptomole range (1 zeptomol ≈ 600 molecules; *SI Appendix*, Figs. S8 *D* and *E* and S9 *A–C*, and Dataset S8). Specifically, we monitored peptides of about 30 proteins identified as part of the TNF-RSC in Fig. 2 and quantified their migration behavior in the two conditions (±DUB). Due to the low abundance of the monitored analytes, in a few instances, proteins could be quantified only in one of the two conditions (*SI Appendix*, Fig. S9*B*). We found that undigested TNF-RSC mostly remained trapped in the well, as evidenced by the stark accumulation of the signal in the first fractions of the gel, indicating that the isolation preserved the signalosome integrity and that its size exceeds the resolving power of the gel (∼1.5 MDa; Fig. 4 *A* and *B*, red trace; Fig. 4*D*; and *SI Appendix*, Fig. S10*A*; see *Materials and Methods* for details about signal normalization and *SI Appendix, Detailed interpretation of the AP-BNPAGE-MS experiment results*). In contrast, USP21 digestion of the isolated TNF-RSC significantly improved its detectability (Fig. 4 *A–C*, blue trace), reduced the signal accumulated in the first fractions relative to the rest of the gel and gave rise to a strikingly periodic signal distribution (Fig. 4*E*). This distribution indicated that several complexes, especially the membrane-proximal core components, the ubiquitin ligase complex LUBAC and the kinase complex IKK, occurred at regularly spaced intervals and with roughly constant intensity ratios across high–molecular weight peaks (Fig. 4*E* and *SI Appendix*, Fig. S10 *B* and *C*). Overall, besides recapitulating the ubiquitin chain dependency of signalosome integrity and providing a lower boundary to estimate its size, these results point at a regular arrangement of its constituting complexes. In this way, the information provided by the BNPAGE experiment provides a useful proof of concept to detect and quantify very low-abundance analytes after immunoprecipitation (IP) and the separation of native complexes.

### Third and Fourth Layers of the Contextual Model: Time-Resolved Stoichiometric Measurements of the TNF-RSC Following Stimulation, Using an AP-AQUA-MS Approach.

To model values for the stoichiometry of the endogenous TNF-RSC complexes, we used three distinct sources of data (*SI Appendix*, Fig. S11*A*). 1) First, we performed absolute quantification of affinity-purified TNF-RSCs over time after TNFα stimulation (5, 10, and 15 min). To this end, we used a targeted PRM MS data acquisition method combined with spike-in of 113 reference peptides of known amounts (AQUA) (*SI Appendix*, Fig. S11 *B–D*, and Dataset S9). For each time point and complex, we plotted the intensity ratio of the complex members relative to the most abundant component (Fig. 4*F* and Dataset S9). 2) Next, we generated an additional stoichiometry estimate by applying the intensity based absolute quantification (iBAQ) algorithm on the data presented in Fig. 2*B* (fractionated AP-MS of the TNF-RSC acquired in DDA mode). This estimate is based on 558 peptides covering 30 proteins with up to 44 peptides/protein (*SI Appendix*, Dataset S9). 3) Finally, we generated a manually curated database of over 100 structural and biophysical publications that report information about size and/or stoichiometry of the TNF-RSC components (*SI Appendix*, Fig. S11*E*, and Dataset S10). This also includes three proteome-wide SEC-MS datasets, which are well suited to provide information about complex isoforms and their approximate molecular weight (16–18). The aim of this database is to evaluate how our data compare to previous findings and how it can reconcile seemingly contradictory observations reported in the literature but also to provide information that cannot be extracted from our ensemble MS measurements (e.g., the homotrimerization of TNFR1 and the homodimerization of TBK1 and CYLD). On a general level, we found that AQUA and iBAQ-based values are in good agreement (*SI Appendix*, Fig. S12 *A–C*) and that stoichiometries are remarkably stable over time poststimulation (Fig. 4*F*). Specifically, we grouped our findings in three classes: 1) ex vivo approximations of in vitro results, 2) controversy-resolving findings, and 3) other novel findings (a detailed discussion is reported in *SI Appendix*, *Extended* Materials and Methods and *Detailed interpretation of the stoichiometry of the TNF-RSC complexes. Absolute stoichiometry of the core complex*). 1) Our data provide an ex vivo estimate of the stoichiometry of the core signaling complex consisting of TNFR1, TRADD, RIPK1, TRAF2, and BIRC2/3. Based on iBAQ data, TRADD, RIPK1, and TRAF2 occur in an approximate 1:1:1 ratio or three copies each (Fig. 4*G* and *SI Appendix*, Fig. S12 *D* and *E*). AQUA measurements for the core complex indicated a different stoichiometry for TRADD, possibly due to specific biases in the selected TRADD peptides. Finally, BIRC2 was consistently found at an average ∼1:4/5 ratio with TRAF2, compared to a 1:3 ratio observed in vitro (19). Similarly, CYLD and SPATA2 are found to stably occur in an ∼1:1 relative stoichiometry, confirming their previous characterization (20). Based on our data, the best fit for LUBAC is a (1 _HOIP_:1 _HOIL-1_:1_SHARPIN_) + (1 _HOIL-1/SHARPIN_) stoichiometry, where excess HOIL-1/SHARPIN subunits are added to a core isostoichiometric complex. This is compatible with crystallographic and biophysical evidence for an isostoichiometric arrangement of LUBAC (1:1:1 or 2:2:2) (21, 22), by the existence of partial complex isoforms and by the potential dimerization of SHARPIN via the SH domain (23). 2) The stoichiometry of the kinase complex IKK (NEMO:IKKA:IKKB) has been extensively investigated in vitro, and dozens of potential solutions are compatible with the current data (24–31). Our results, in combination with 1) absolute measurement of A549 cytosolic pools (*SI Appendix*, Fig. S14*A*), 2) bioinformatic analysis of tissue-level data (*SI Appendix*, Fig. S14*B*), and 3) evidence of distinct IKK isoforms from SEC-MS experiments (*SI Appendix*, Fig. S12*H*), support a model where the number of NEMO molecules in complex duplicates upon TNF-RSC recruitment, associating with two (or possibly more) distinct isoforms of the IKKA/IKKB (Fig. 4*H*). Importantly, this model reconciles and provides a rationale for several seemingly contradictory reports (*SI Appendix* and Dataset S10), even though the exact ratios of IKKA/IKKB cannot be conclusively extracted from our data. 3) Finally, our data indicate an approximate 2_TAB1_:2_TAB2_:1_TAK_ ratio for the kinase complex TAB/TAK1 and a relative stoichiometry of 1_TANK_:1_TBK1_ for the kinase TBK1 complex (*SI Appendix*, Fig. S12 *D* and *E*), with a proposed absolute stoichiometry of 2_TANK_:2_TBK1_ based on the known dimerization of TBK1 and crystallographic evidence of TBK1 tetramerization with AZI2 (32). Overall, by using a combination of MS-based ensemble measurements and literature mining, we present here hypotheses about the stoichiometries of all the main complexes of the TNF-RSC. While we have observed heterogeneities in such stoichiometries (such as, for instance, the duplication of the scaffold subunit of the IKK complex), targeted studies will be required to more comprehensively illuminate such variations. Furthermore, because of technical variability, complexes isoforms with similar ratios, as well as large ratios, cannot be reliably captured by our MS data, as also highlighted by the discrepancies between AQUA and iBAQ measurements. Finally, we report here that both WHIP and UBASH3B are largely substoichiometric with respect to other core components of the TNF-RSC, and their abundance (2 to 3%) compares to proteins that have no structural role in the assembly of the TNF-RSC, such as A20 (*SI Appendix*, Fig. S12*F*). This is in line with the absolute quantification we have performed on UBASH3B isolates (Fig. 3*F*), as well as with the observation that genetic ablation of the phosphatase does not seem to destabilize the signalosome (*SI Appendix*, Fig. S7 *A* and *B*). Because our time course analysis provides information about association dynamics as well as ratios, we describe here also the association profiles of the monitored TNF-RSC members (*SI Appendix*, Fig. S13*A*, and Dataset S9). Based on unsupervised hierarchical clustering, we grouped the proteins in three classes and found that their association profiles broadly reflect the known organization of the TNF-RSC complexes, as can also be appreciated by principal component analysis of the same profiles (*SI Appendix*, Fig. S13*B*). In summary, by combining differential AP-BNPAGE-MS and stoichiometry measurements, we provide an absolute quantitative, ex vivo, time-resolved ensemble description of the TNF-RSC size, ubiquitin regulation, and complex stoichiometries.

### Fifth Layer of the Contextual Model: Resource Allocation of the TNF-RSC Using the Combination of AP-AQUA-MS and Lysate-AQUA Analysis.

**Fig. 5.**
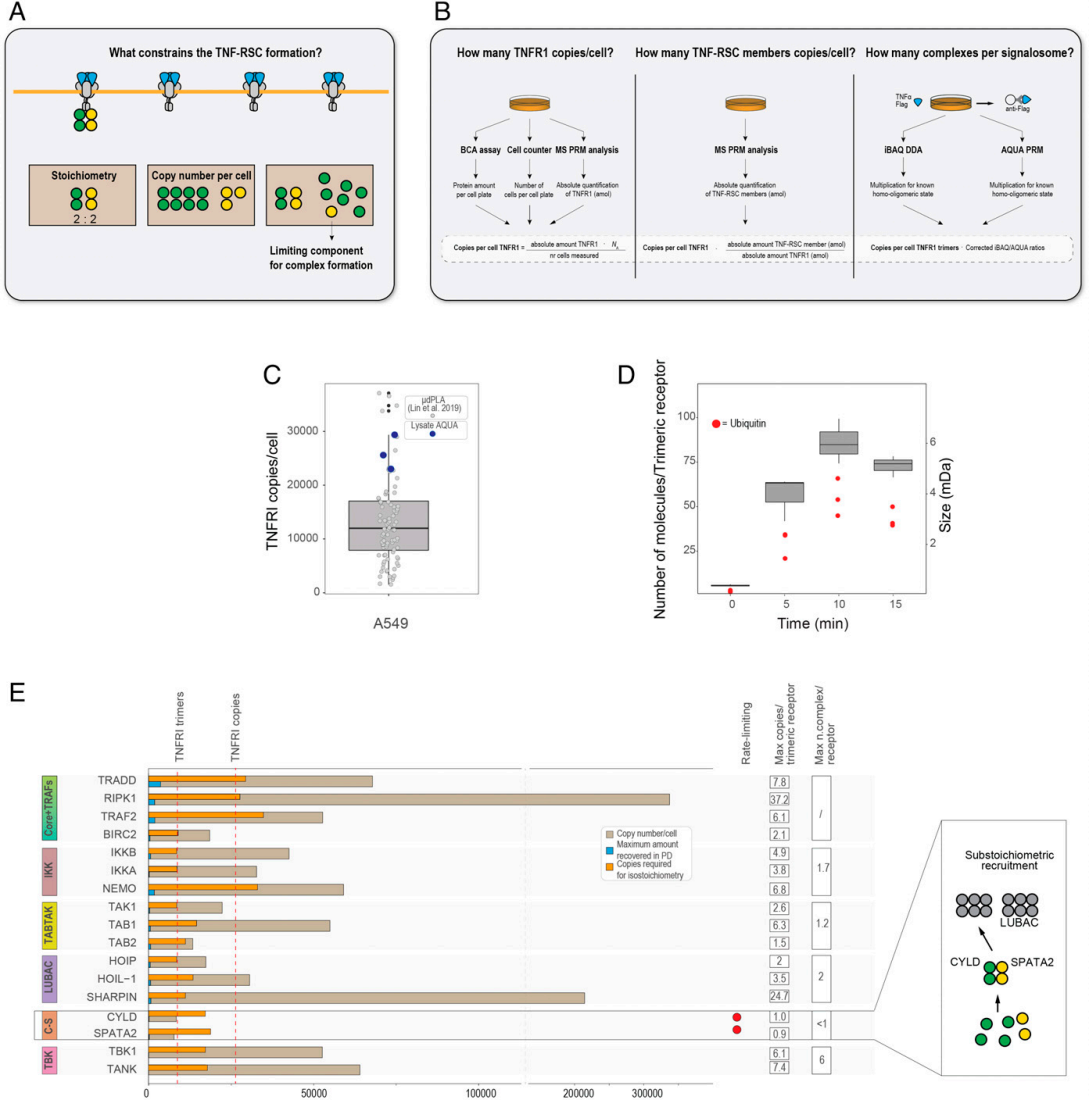
Cellular constraints on TNF-RSC formation. (*A*) Design: estimate of copy number/cell is combined with knowledge about stoichiometry to define receptor occupancy and identify limiting complex components (yellow elements). (*B*) Workflow for the calculation of proteins which constrains the formation of TNF-RSC. Briefly, total protein amount in the cell is calculated by PRM assay; this amount is normalized for the cells used in the experiment. Stoichiometry ratio obtained from iBAQ and AQUA dataset normalized for the copy per cell of TNFR1 reveals the number of complexes per signalosome. Combination of signalosome stoichiometry and the copies per cell in the lysate provides the limiting component for the complex formation. (*C*) Box plot displaying lysate estimates of the number of molecules per cell of TNFR1 (blue dots) and the number determined in a nominally identical cell line using PLA (gray dots) (33). (*D*) MW and number of molecules of the TNF-RSC over time as estimated by AP-AQUA-MS. Red dots indicates estimated number of ubiquitin molecules. (*E*) Resource allocation plot. Number of copies per cell (gray bars) are compared with estimated copies isolated from affinity-purified TNF-RSC (blue bars) and the number of copies required to achieve 1:1 stoichiometry with the receptor (orange bars). Data (reported in *SI Appendix*, Dataset S12) are generated from the stoichiometry calculated in the iBAQ dataset.

In the last step of our workflow, we set out to model the abundance and formation of the TNF-RSC in the context of the cell. How many receptors are present on average in a cell? How many complexes are going to associate with them? What constrains the signalosome formation? We reasoned that if we estimated the number of copies per cell of all the TNF-RSC members and combined this information with the determined stoichiometries for the TNF-RSC complexes, we should be able to pinpoint those components that are limiting for the formation of the signalosome. For instance, consider a complex *C* with subunits *X* and *Y* in a 2:2 stoichiometry (Fig. 5*A*). If protein copies*_Y_* < protein copies*_X_*, *Y* will be limiting for the formation of complex *C* but also for the complete formation of the signalosome that complex *C* associates with. Fig. 5*B* illustrates how the relevant quantities have been calculated, and further details are reported in *Materials and Methods* and Dataset S12. We first performed an experiment to estimate the copy number per cell of TNFR1 receptors in A549 cells. To this end, we quantified TNFR1 using targeted MS (PRM) in combination with three synthetic peptides of known amounts as a reference, which we spiked in after cell lysis to correct for potential loss during digestion. We then normalized the thus estimated TNFR1 absolute amount by the number of cells used in the experiment (Fig. 5 *B*, *Left*). Finally, we benchmarked our results against a recently published estimate carried out in A549 cells using an orthogonal and accurate method, proximity ligation assay (PLA) (Fig. 5*C*) (33), indicating that our estimate is in the high range of what had been previously determined. Next, we set out to estimate the number of protein copies per cell of the other TNF-RSC members. To do that, we performed absolute quantification on the 12 lysates that have been used for the AP-AQUA-MS experiment (*SI Appendix*, Fig. S14*A*). For these experiments we utilized the TNFR1 as a calibrant to translate absolute amounts in protein copies per cell (Fig. 5 *B*, *Center*). We asked how representative our cell line is and compared our values with the deep proteome profiling of 29 human tissues (34). We found them to be overall in good agreement (*r* = 0.769; *SI Appendix*, Fig. S14 *B* and *C*) and relatively stable around the mean of the proteome-wide abundance values (*SI Appendix*, Fig. S14*D*). Next, we used the AP-AQUA-MS data to calculate a lower boundary in the size of the TNF-RSC, by estimating the average number of molecules (and their summed molecular weight [MW]) associated with the receptor (Fig. 5*D* and *SI Appendix*, Fig. S14 *E* and *F*, for absolute amount and recovery yield of TNF-RSC members in AP-AQUA-MS experiment). We found that the signalosome size peaks at 10 min reaching an estimated molecular weight of about 6 MDa, a result that is compatible with the AP-BNPAGE-MS data, with ubiquitin making up the majority of its mass. Finally, to identify those proteins that are limiting for the formation of the complex, we calculated the copies of proteins needed to form a complete signalosome (i.e., having at least one copy of each complex) based on the ratios obtained from iBAQ and AP-AQUA-MS data separately, combined with basic assumptions from the literature (TNFR1 is trimeric; CYLD and TBK1 are dimeric) (Fig. 5 *B*, *Right*). This information is displayed in two different plots in Fig. 5*E* (using iBAQ dataset) or *SI Appendix*, Fig. S14*G* (using AP-AQUA-MS dataset). In these plots, the cellular copy number of the TNF-RSC members (gray bars) is compared with the theoretical copy number needed to occupy all the receptors given the determined stoichiometries (orange bar). The blue bar in Fig. 5*E* and *SI Appendix*, Fig. S14*G*, indicates the estimated recovered amount of TNF-RSC members from the AP-AQUA-MS. Our data consistently identify CYLD-SPATA2 as limiting members (Fig. 5*E* and *SI Appendix*, Fig. S14*G*, red dot), and, on average, indicate that most complexes are present in less than one or at most one copy per receptor, even at the peak of recruitment (10 min). Finally, we asked whether this systems-level data allow us to make predictions about the composition of the signalosome and the effect of protein overexpression. To do this, we focused on CYLD-SPATA2, which is substoichiometric with respect to the TNFR1 receptor. Since we know that 1) CYLD and SPATA2 are bound in a 2:2 stoichiometry (Fig. 4*F* and *SI Appendix*, Fig. S12 *D* and *E*) (20), 2) endogenous CYLD and LUBAC components are more abundant than SPATA2 in the proteome profiling of 29 human tissues (*SI Appendix*, Fig. S14 *A* and *B*), and 3) SPATA2 mediates CYLD recruitment to LUBAC (7, 20), we would predict that only part of the cytoplasmic pool of CYLD is bound to SPATA2 and that as a consequence, CYLD-SPATA2 complex is substoichiometrically bound to the LUBAC complex. In keeping with this, our iBAQ analysis indicates that only ∼20 to 25% of LUBAC molecules are bound to CYLD-SPATA2 (*SI Appendix*, Fig. S12*D*). Likewise, endogenous abundance of the many paralogs/redundant subunits present in the system (TRAF2/TRAF5, TRAF2/TRAF1, BIRC3/BIRC2, TAB2/TAB3, and TBK1/IKKE) is enough to crudely predict their receptor-bound abundance, even though more detailed mechanistic knowledge is essential to understand their differential recruitment/activity mechanisms. Overall, we propose a framework to combine protein interaction data with estimates of absolute protein amounts to contextualize PPIs inside the cell and define some of the constraints regulating their formation.

## Discussion

In this study, we apply an integrated MS-based interactomic pipeline, which mediates the transition from a generic, decontextualized identification of PPIs toward the generation of a quantitative PPI model in the context of specific cell states. The presented strategy alleviates several limitations of current proteomic approaches, specifically the sensitivity, quantification accuracy, and combination of data from different sources, and is based on the complementary contribution of several orthogonal techniques and acquisition methods. They each defined, layer by layer, the composition, assembly organization, stoichiometry, and PTM and signaling dependence, as well as systems properties of a target assembly. As applied to the TNF-RSC, this workflow has provided glimpses into its composition, regulation, temporal organization, and system-level constraints. With the first step of the workflow, we combined different MS acquisition modalities and two orthogonal controls to map the TNF-RSC composition. Of the several interesting candidates we have thus nominated, we confirmed the interaction with WHIP and carried out a more extensive characterization of UBASH3B since phosphatases have been comparatively poorly characterized in the context of TNF signaling, and thus, we suggested a new layer of signaling regulation. In the second and third steps, we resolve along the MW dimension and in time important architectural features of the TNF-RSC, information that can be readily incorporated in integrative structural models. The differential AP-BNPAGE-MS analysis, with some technical caveats describe above, provides a unique dimension to understand the architecture of the TNF-RSC, and its results show that the determined interactome is organized in a highly modular fashion, as expected from the ubiquitin dependency of its assembly. It is important to bear in mind that the analysis of these data remains at present confounded by several factors, including disassembly of the signalosome and limit of gel resolution and in the identification and quantification of very low-abundance TNF-RSC components (low femtomoles range), which can result in migration patterns not consistent with their known MW and leads to results which could be difficult to interpret. It must also be stressed that at present, at least for low-abundance proteins, performing BNPAGE on isolates requires substantial experimental effort and cellular amounts. Indeed, this is an example of an endogenous membrane complex being separated on a fractionation dimension in combination with targeted MS to separate protein subcomplexes in the low attomoles range, but we expect that such experiments will be routinely incorporated in interactomic studies as they will become increasingly less time-consuming and labor-intensive. In a similar vein, there are only few publications that have used bottom-up MS to decode the absolute stoichiometry of specific complexes (35, 36). Despite the challenges entailed, we show that stoichiometry determination on pulldown samples represents a unique vantage point to capture ensemble, structural information locally (pulldowns) and globally (lysate). This information is essential to interpret and validate in vitro data but also to provide novel, self-contained, and robust insights. Remarkably, we could capture instances of variable stoichiometries, such as the proposed duplication of the scaffold subunit of the IKK complex, NEMO. However, because MS data rely on ensemble measurements and because such measurements suffer from a certain technical variability, other instances of stoichiometry heterogeneity, if present, may remain undetected. We also encountered examples of discrepant ratios (iBAQ vs. AQUA), which impede providing unique solutions for all stoichiometries. Finally, on a systems level, at least two seminal studies have examined the role of protein abundance in other signaling systems (37, 38) but have not taken into account the specific structural and stoichiometric requirements that proteins need to meet in order to be functional. We show here that by combining knowledge about stoichiometry and absolute abundances in the isolated signalosome and lysates, we can propose boundaries in the composition and formation of the signalosome in question and make educated predictions on recruitment stoichiometries. Given the nature of our data, our model is limited in least two ways: it needs to include assumptions derived from the literature about, e.g., the homooligomerization of TNFR1, and it does not consider complex effects due to competitive binding and expression of homologs. To conclude, one of the greatest challenges of current biology is the modeling of the proteome in context, and interactomic studies have much to contribute to this endeavor. However, their use will remain limited unless they will incorporate quantitative, context- and structure-informed aspects. Our study describes a platform to achieve such integration.

## Materials and Methods

### Cell Culture and Cell Lines Generations.

#### Cell culture.

Cells were cultured in DMEM supplemented with 10% Fetal Calf Serum (FCS; Bioconcept) and 50 μg/mL of both Penicillin and Streptomycin (Sigma-Aldrich). Trypsin-EDTA (Life Technologies) was used for propagation, and frozen stocks were prepared in DMSO:FCS (1:9).

#### Generation of UBASH3B and LUBAC Strep-HA stable cell line.

UBASH3B and LUBAC expression plasmids were generated by enzymatic LR clonase reaction (Invitrogen) of a UBASH3B pDONR vector (Orfeome v5.1) and a pTO-SH entry vector, encoding a C- or N-terminal Strep-HA tag. The UBASH3B expression vector and pOG44 vector (Invitrogen) were cotransfected in HEK Flp-In 293 T-Rex cells (Invitrogen) using X-tremeGENE transfection reagent (Invitrogen). Two days after transfection, cells that had undergone recombination were selected using DMEM supplemented with 100 μg/mL of hygromycin (Invitrogen) and 19 μg/mL of blasticidin (Huberlab) for 2 to 3 wk.

#### CRISPR/Cas9-mediated gene KO of UBASH3B and WHIP.

Optimized CRISPR Design web tool (crispr.mit.edu) was used for the selection of the target sequences and the corresponding CRISPR guide RNAs (gRNAs). DNA oligonucleotides containing the gRNA sequence were annealed and then cloned into the hSpCas9 plasmid (pX458, Addgene) using BbsI restriction sites. For the generation of the UBASH3B, KO A459 cells were transfected with two hSpCas9 constructs which encode a GFP marker and gRNAs that target the third and fourth exon of the gene. The deletion of WHIP in A459 cells was performed by a gRNA pair that targets the first exon. Four hours after the transfection the media was replaced, and cells were recovered for 72 h. FACS sorting was performed with 1 × 10e6 cells resuspended in PBS containing 1% FBS. Transfected cells expressing the GFP marker were isolated by FACS (BD Facs Aria IIIu sorter), and single cells were sorted into a 96-well plate. The sorted cells were expanded for several weeks until cell colonies were formed. Successful deletion events were detected by Western blot analysis using specific antibodies against UBASH3B and WHIP. The gRNA target sequences and oligonucleotides are reported in the Resource table.

### Protein Purification and Affinity Purifications.

#### Purification of the cytokine TNFα.

*Escherichia coli* (BL21) was grown in LB medium overnight at 37 °C with shaking and diluted 1:10, and the expression of a GST-Flag–tagged version of the ligand was induced with 0.05 mM IPTG after 1 h for 4 h. Cells were harvested and lysed using B-PER Bacterial Protein Extraction Reagent (Thermo Fisher Scientific) supplemented with DNase (Roche) and protease mixture (Roche). Debris was removed by high-speed centrifugation and the lysate incubated overnight with preequilibrated Glutathione Sepharose beads (Sigma Aldrich; 1.5 mL slurry per liter of culture). Beads were washed with a total of 20 to 30 volumes of PBS and Cleavage buffer (50 mM Tris, pH 7, 150 mM NaCl, 1 mM EDTA, 1 mM DTT) and incubated with PreScission Protease (GE Healthcare) overnight. Supernatants were collected, if needed concentrated to at least ∼1 mg/mL, and separated onto a Superdex S75 10/300 (GE Healthcare). Fractions corresponding to the trimeric cleaved protein peak were collected, and protein purity was verified on an sodium dodecyl sulfate polyacrylamide gel electrophoresis (SDS-PAGE) gel (NuPAGE Bis-Tris gels, Thermo Fisher Scientific) and used for subsequent experiments.

#### Purification of UBASH3B antibodies.

To perform endogenous purification, we designed a custom polyclonal antibody against the N-terminal region of UBASH3B (aa 2 to 16; sequence CAQYGHPSPLGMAARE). The following parameters were considered for the peptide choice: 1) exposition and lack of secondary structure [based on Psipred analysis (39)], 2) low sequence homology with other human proteins, 3) no reported PTMs or protein interactions, and 4) stability in solution (we used ProtParam Tool from Expasy to monitor the stability). The peptide was synthetized, coupled to KLH carrier protein, and used for rabbit immunization with the Speedy 28-Day program by Eurogentec. The final bleed was affinity-purified using an AKTA pure chromatographic system (GE Healthcare) with the epitope antibody column using a running buffer containing 50 mM Hepes (pH 7.5), 150 mM NaCl, and 0.1 M Glycine (pH 3) for the elution. The column for the affinity purification was prepared coupling the peptide CAQYGHPSPLGMAARE to *N*-Hydroxysuccinimide group of HiTrap NHS-Activated affinity column (GE Healthcare). Eluate was neutralized in Tris base solution 0.1 M, pH 8.8, dialyzed overnight in buffer 50 mM Hepes (pH 7.5), 150 mM NaCl using membrane dialysis tube (Pur-A-Lyzer Mega Dialysis 3500 KDa) (Thermo). The dialyzed eluate and the flow-through obtained from peptide affinity purification were quantified and coupled to protein A Sepharose 4 Fast Flow (GE Healthcare) following the protocol in ref. 40. Briefly, 10 mg of specific and nonspecific antibodies were incubated with 5 mL of wet protein A beads for 1 h, beads were extensively washed with 0.2 M Sodium Borate, pH 9, and cross-linked with 20 mM of DMP for 1 h. After quenching reaction with ethanolamine 0.2 M, beads were aliquoted (∼200 µg of antibody per purification) and ready to use.

#### TNF-RSC affinity purification.

For DDA (A549 WT and KOs) and PRM datasets, 6 × 15 cm dishes of confluent A549/replicate were stimulated (DDA, continuous stimulation; PRM, pulsed stimulation, 1 min) with ∼800 ng/mL purified flag-tagged TNFα; cells were washed two times with ice-cold PBS, and ∼1 mL lysis buffer (HN Buffer supplemented with 10% Glycerol, 2 mM EDTA, 0.5% Nonidet P-40/IGEPAL, 400 μM Sodium Orthovanadate, protease inhibitors, 10 μM PR619 [Abcam]) was added to each dish before cell collection with a cell scraper. Lysis was carried out for 20′ at 4 °C on an orbital rotor, and debris was removed by centrifugation at 14,000 × *g* on a tabletop centrifuge at 4 °C. Supernatants were incubated from 4 h to overnight with AntiFlag M2 affinity gel (Sigma-Aldrich). Beads were subsequently washed two times with lysis buffer and three times with HN Buffer (50 mM Hepes, 150 mM NaCl, pH 7.4), before elution with Urea (8M). For control “Unstimulated,” cell lysates were incubated with Flag-tagged TNFα overnight, while for control “His_TNFα,” cells were stimulated with His-tagged TNFα and processed as described in the following paragraph. For the AP-BNPAGE-MS experiments, 50 × 15 cm and 66 × 15 cm dishes were used for the −DUB and +DUB conditions, respectively; TNF-RSC was purified as described before; and the elution of the TNF-RSC from the beads (5 mg/mL 3xFlag peptide [Biotrend]) was coupled in the latter condition with USP21 treatment (0.5 μM; Boston Biochem).

### MS Sample Preparation.

#### TNF-RSC AP-MS.

Affinity-purified samples in Urea (8M) were reduced with 5 mM TCEP (30 min at 37 °C) and alkylated with (10 mM IAA at room temperature). Urea was diluted to a concentration of 5.5 M for Lys-C proteolysis (Wako) (0.4 µg, 3 h) and trypsin proteolysis (Promega) (1.2 µg, overnight). Digested samples were acidified by addition of formic acid, cleaned up with microspin columns (The Nest Group), and subjected to high pH fractionation, with a procedure based on the high pH fractionation kit by Thermo.

#### UBASH3B AP-MS.

A 6 × 15 cm dish/replicate of Flp-In HEK293 cell line expressing UBASH3B in an inducible manner was used; protein expression was induced by addition of doxycycline (Sigma) at 1.33 μg/mL for 24 h prior to harvesting. Cell pellets were resuspended and lysed in 4 mL HNN lysis Buffer (50 mM Hepes, pH 7.5, 150 mM NaCl, 50 mM NaF, 0.5% Igepal CA-630) supplemented with 400 nM Na_3_VO_4_, 1 mM PMSF (Sigma-Aldrich), 1.2 μM Avidin (IBA Lifesciences), and 1× Protease Inhibitor mix (Sigma) and incubated on ice for 10 min. Debris was removed by centrifugation at 14,000 × *g* for 20 min at 4 °C on a tabletop centrifuge, and the supernatant was incubated with cross-linked beads (5 mM disuccinimidylsuberate [DSS], Thermo), in 50 mM Hepes (pH 8.0), 150 mM NaCl for 30 min at 37 °C with strong agitation and quenched with 50 mM ammonium bicarbonate for 30 min at 37 °C with strong agitation. Beads were washed two times with lysis buffer and three times with HNN buffer (50 mM Hepes [pH 7.5], 150 mM NaCl, 50 mM NaF); beads and bound proteins were transferred in 10-kDa molecular weight cutoff spin column (Vivaspin 500, Sartorious) for proteolysis. Briefly, beads in solution were centrifuged at 8,000 × *g* until dryness. Samples were denatured, reduced (8 M Urea and 5 mM TCEP in 50 mM ammonium bicarbonate, 30 min), and alkylated (10 mM iodoacetamide, 30 min). Each sample was subsequently washed three times by flushing the filter with 25 mM ammonium bicarbonate and proteolyzed with 0.5 μg of Trypsin (Promega, sequencing grade) for 16 h at 37 °C with agitation. Proteolysis was quenched with 0.1% TFA, and peptides were purified with a C18 microspin column (Nest Group) and dried before being resuspended in 20 μL 0.1% formic acid and 2% acetonitrile. Indexed Retention Time (iRT) peptides (Biognosys) were spiked in each sample (1:50) before LC-MS/MS analysis for quality control.

#### LUBAC AP-MS.

For affinity purification Flp-In HEK293 cell lines with stably integrated *N*- or C-terminal tagged HOIL-1, HOIP or SHARPIN were used. The expression of the Strep-HA tagged bait proteins was induced by addition of 1.33 µg/mL doxycycline (Sigma) for 24 h prior to harvesting. Per replicate, three 150 mm tissue culture plates were harvested, and cell pellets were lysed in 4 mL HNN lysis Buffer (50 mM Hepes, pH 7.5, 150 mM NaCl, 50 mM NaF, 0.5% Igepal CA-630) supplemented with 400 nM Na_3_VO_4_, 1 mM PMSF (Sigma-Aldrich), 1.2 µM Avidin (IBA Lifesciences), and 1× Protease Inhibitor mix (Sigma) and incubated on ice for 10 min. Cell debris was removed by centrifugation at 14,000 × *g* for 20 min at 4 °C, and the supernatant was incubated with Strep-Tactin beads (IBA LifeSciences) for 1 h at 4 °C. Beads were washed two times with lysis buffer and three times with HNN buffer (50 mM Hepes [pH 7.5], 150 mM NaCl, 50 mM NaF). Affinity-bound proteins and protein complexes were eluted from Strep-Tactin beads with 2mM biotin (600 µL). Proteins were precipitated in trichloroacetic acid solution (25%), and upon washing with acetone the dried pellet was dissolved in 8 M urea. After reduction [5 mM Tris(2-carboxyethyl)phosphine] and alkylation (10 mM iodoacetamide), proteins were digested with 0.8µg trypsin (Promega, sequencing grade) for 16 h at 37 °C under agitation. The peptide clean-up was performed with a C18 microspin columns (Nest Group). Peptides were dried in a speedvac and resuspended in 20 µl 0.1% formic acid and 2% acetonitrile.

#### UBASH3B IP-MS.

A549 were grown in 10 × 15 cm tissue culture plates at 80% confluency, harvested and the cell pellets were snap-frozen. The frozen pellets were lysed in 8 mL of lysis buffer (0.5% Nonidet P-40, 50 mM Hepes pH 7.5, 150 mM NaCl, 50 mM NaF, 400 nM Na_3_VO_4_ supplemented with 1mM PMSF and protease inhibitor mixture (P8849, Sigma)). The lysates were incubated on ice for 20 min. The cleared cell lysate was incubated with protein A beads coupled with antibody raised against a UBASH3B N-terminal peptide overnight on a circular rotor. After incubation, beads were washed and proteolyzed following on bead digestion protocol as described in UBASH3B AP-MS.

#### Proteome profile.

Unless otherwise stated, lyophilized samples were resuspended in 8 M Urea in 50 mM ammonium bicarbonate, reduced with 5 mM TCEP (Sigma-Aldrich) for 30 min at 37 °C on a thermomixer (Eppendorf) and subsequently alkylated with 10 mM IAA (Sigma) at room temperature in the dark. Urea was first diluted to a concentration of 5.5 M and Lys-C (Wako) at a 1:100 wt/wt ratio was added for 2.5–3 h at 37 °C. Subsequently, urea was diluted to 1 M and samples were further digested by addition of trypsin (Promega) at 1:50 wt/wt ratio overnight at 37 °C. Digested samples were acidified by addition of formic acid and purified using either SPE cartridges (Waters) or microspin columns (The Nest Group). Eluates were dried, resuspended in acetonitrile 2–5% and formic acid 0.1%, typically with addition of iRT peptides (Biognosys), sonicated and centrifuged for 5–10 min at 10,000 *g* before MS injection.

#### AP-BNPAGE-MS.

TNF-RSC complexes were isolated from ∼1 × 10e9 and ∼1.3 × 10e9 A549 cell. Forty μL of eluted complex was supplemented with 12 μL of Native Sample Loading Buffer (Invitrogen) and loaded on a NativePage 3 to 12% Bis-Tris precast protein gel (Invitrogen) for native separation, according to the manufacturer’s instructions, with the following exceptions: cathode chamber was filled with light blue cathode buffer, and BNPAGE was running for 3 h at 4 °C with a three-step gradient (150 to 180 to 200 V). Once the run was finished, proteins were stained with SimpleBlue SafeStain (Invitrogen) and proteolyzed following proteaseMAX surfactant (Promega) in-gel digestion protocol. To excise 64/76 bands with the same size from a native gel preparation, a custom-designed device containing ∼100 parallel blades spaced 1 mm from one another was used. Briefly, excised protein bands were destained, dehydrated, reduced, and alkylated before proteolysis. Digestion was performed in 50 μL digestion solution (0.5 μg of trypsin [Promega], sequencing grade; 0.1 μg Lys-C [Wako]; 0.01 proteaseMAX surfactant [Promega] in 50 mM ammonium bicarbonate). After overnight digestion, peptides were collected while gel bands were covered with 50% acetonitrile solution for 30 min. Peptide solutions generated from the proteolysis and from the treatment of gel bands with 50% acetonitrile were dried and resuspended in 10 μL 0.1% formic acid and 2% acetonitrile.

### MS Data Acquisition and Analysis.

All acquisition and basic data processing parameters are described in *SI Appendix*, *Extended* Materials and Methods.

### Biochemical and Cellular Assays.

#### Protein and peptide concentration measurement.

Protein amounts from cleared lysates were measured using a Bicinchoninic acid assay (BCA) assay kit (Pierce) following the manufacturer’s instructions. Peptides amounts were determined using a Quantitative Colorimetric Assay Kit (Pierce) following the manufacturer’s instructions.

#### DEVDase assay.

The assay was performed as previously described (41). Briefly, A549 cells were transfected with siRNA against UBASH3B or an Alexa488-labeled control (QIAGEN) 48 h before the indicated treatment. After the treatment, the medium was removed by aspiration, and 50 μL of 1% DISK lysis buffer (20 mM Tris⋅HCl, pH 7.5, 150 mM NaCl, 2 mM EDTA, 1% Triton X-100, 10% Glycerol) was added to each well. After incubation for 20 min at room temperature, 450 μL of DEVDase assay mix (20 μL Ac-DEVD-AMC [Sigma], 1 mM DTT, 25 mM Hepes, pH 8.0) was added to the lysate in each well. The plates were incubated at room temperature, and the DEVDase activity was measured for 13 h every 30 min.

#### siRNA transfection.

A549 were grown to 60 to 80% confluence in a 24-well plate, and transfection was carried out as follows: two solutions of 25 μL Opti-MEM medium and 1 μL Lipofectamine RNAiMAX (Thermo Fisher) or 1.5 μL of siRNA (10 μM, QIAGEN) were prepared independently and subsequently mixed and incubated at room temperature for 5 min. Cells medium was replaced by 450 μL of fresh medium, and the transfection mix was added to the cells to a final siRNA concentration of 20 nM. Incubation with siRNA was carried out for 48 h.

### siRNA Transfection for Cell Viability Assay.

HT1080 cells were transfected for 48 h with 20 nM siRNA SMARTpools (Dharmacon) targeting human *Ubash3b* (no. L-008533-00-0005), *Whip1* (no. L-010072-01-0005), and *Hoip* (no. L-021419-00-0005). Nontargeting siRNAs were used as controls (nontargeting 1, no. D-001810-01-05; nontargeting 2, no. D-001810-02-05). Transfection was achieved using Lipofectamine RNAiMAX (Thermo Fisher) as per manufacturer’s instructions. siRNA knock-down was confirmed by Western blot analysis using anti-UBASH3b (Abcam, ab34781), anti-WHIP1 (Abcam, ab4731), and anti-HOIP (Bethyl Laboratories, A303-560A) antibodies.

### Cell Viability Assay.

A total of 5 × 10^3^ HT1080 cells were seeded per well of a 96-well plate, overnight. Indicated siRNA were delivered for 48 h, before overnight treatment with DMSO, 10 ng/mL TNF (Enzo), and 100 nM SM164 (Insight Bio) individually or in combination. Viability was then assessed by CellTiter-Glo assay (Promega) according to manufacturer’s instructions, using a Victor ×5 HTS microplate. Graphs and statistical analysis were performed using GraphPad Prism V9.0. Standard two-way ANOVA tests were used to assess statistical differences in viability assays.

#### Western blot.

Samples were mixed at 1:2 ratio with Laemmli Buffer and boiled for 10 min at 95 °C. For the WB related to UbiCrest assays, samples were not boiled. Homogeneous amounts of proteins were loaded on precast NuPAGE 4 to 12% Bis-Tris Protein Gel (Thermo Fisher) and were separated in a MOPS or MES Buffer (Thermo Fisher) at 80 to 150 V. Prior to blotting to a nitrocellulose or PDVF membrane (GE Healthcare), both membranes and gels were equilibrated in methanol/transfer buffer. Semidry transfer was carried out on a Trans-Blot Turbo Transfer System (Biorad). Transfer was verified by Ponceau staining, and membranes were blocked in TBST + 5% milk for 1 h and incubated with the primary antibody in TBST + 4% milk at 4 °C overnight. All antibodies are included in Dataset S13. After overnight incubation, membranes were washed three times for 10 min at 4 °C with TBST and incubated for 1 h with mouse or rabbit secondary antibody. After three washes with TBST for 10 min at 4 °C, excess buffer was soaked, and membranes were developed using an ECL kit (GE-Healthcare) and the signal detected using a Fusion FX imager (Vilber).

### TNFR1-SC1 Purification.

HT1080s were grown to 100% confluency before treatment with warmed DMEM (10% FCS and 50 µg/mL Penicillin/Streptomycin) containing 3xFLAG-hTNF (800 ng/mL) for indicated times. Stimulation was terminated by removing media and washing plates with ice cold PBS, before freezing at −80 °C. After thawing, cells were lysed in DISK lysis buffer (20 mM Tris⋅HCl, pH 7.5, 150 mM NaCl, 2 mM EDTA, 1% Triton X-100, 10% glycerol) supplemented with complete protease inhibitor mixture (Sigma), phosSTOP phosphatase inhibitor (Sigma), and 10 µM PR619 DUB inhibitor (2B Scientific). Lysates were extracted and clarified at 4 °C, by sequentially rotating and centrifuging (14,000 rpm) for 20 and 15 min, respectively. For untreated control samples, 800 ng/mL 3xFLAG-TNF was added postlysis. Cleared lysates were rotated overnight at 4 °C in the presence of 20 µL anti-FLAG M2 beads (Sigma). Immunocomplexes were washed four times with DISK buffer supplemented with PR619 (10 µM), before elution with 60 µL glycine (0.2M) for 15 min on ice. Forty µL of eluate was neutralized by adding 8 µL of NH_4_NCO_3_, before boiling in the presence of 1× SDS loading dye.

### Absolute Quantification.

#### Peptide linearity assessment.

An equimolar mix of 113 AQUA peptides was serially diluted in a lysate background, and 0.4, 4, 40, 400, 4,000, and 40,000 amol were injected on a QExactive HF Hybrid Quadrupole-Orbitrap mass spectrometer (Thermo Fisher) and monitored in PRM with the method described in *SI Appendix*, *Extended* Materials and Methods, PRM Data Acquisition TNF-RSC Lysate, with a fill time of 54 ms and resolution of 30,000.

#### AQUA peptides spike-in.

For both lysate and affinity-purified samples, 98 to 113 AQUA peptides were spiked into the final peptide matrix before MS injection. Peptides were spiked in at four (AP-AQUA-MS samples) or five (Lysate-AQUA) different concentrations, so as to approximate the abundance of the endogenous proteins, and with peptides ranging from 16 to 160,000 amol. This spike in strategy has the drawback of potential artifacts related to differential digestion of peptides as well as different physical–chemical properties, leading to potentially biased estimates for some proteins, but it enables matching more closely endogenous and reference peptide concentrations. This bias seems visible in the AQUA-based quantification of TRADD, for which twice as many molecules are estimated as compared to the iBAQ quantification and prior literature knowledge. For the estimation of the TNFR1 copy number, spike-in was performed after cell lysis.

#### Estimation of protein amount per cell.

First, we estimated the number of TNFR1 copies per cell. A549 cells were grown to confluence in 1 × 15 cm dish per replicate for three technical replicates. Cells were trypsined and counted with a Thermo cell counter (Countess II cell counter), resulting in a count of ∼1.8 × 10e7 cells per dish. Lysates (HNN lysate buffer) protein amount was determined by BCA kit (Pierce) to correspond to about 4.1 mg per dish. From this, we estimated an average protein content per cell of ∼230 pg, which is in the range of what has been previously reported for A549 (42). We proteolyzed 100 μg of lysates (which corresponds to about ∼435,000 cells) using FASP-coupled proteolysis (43). Synthetic TNFR1 peptides were spiked in after lysis and before further sample processing to account for potential losses during digestion and C18 cleanup. We estimated that about 0.7% of the sample was injected (corresponding to ∼3,043 cells). We monitored and quantified three TNFR1 peptides (Dataset S11) by PRM; average ratio of endogenous and reference peptides provides the absolute amount of TNFR1. Copy per cell of TNFR1 is estimated dividing the estimated absolute amount with the calculated number of processed cells. Second, we estimated the absolute amount of the TNF-RSC members using spiked-in reference synthetic peptides, as described in *AQUA peptides spike-in*. We performed targeted PRM measurements and estimation of absolute quantities in 12 samples. These samples correspond to the A549 lysates that have been used for the AP-AQUA-MS experiment (three per time point, 0, 5, 10, and 15 min). Next, we translated the absolute amount of the quantified proteins from moles to copies per cell. To do that, we have used as a ruler the TNFR1 copy number per cell that we have calculated in section (i) of this paragraph. Specifically, we multiplied the estimated copies of TNFR1 (∼25,000 per cell) by the absolute abundance of each monitored protein divided by the absolute abundance of TNFR1 from the same experiment.

#### Copies required for isostoichiometry.

Data obtained from iBAQ and AP-AQUA-MS experiments were integrated with basic assumptions from the literature about known homooligomeric states of some TNF-RSC proteins. Given these ratios and the copies per cell of the TNF-RSC proteins, we estimated how many copies of a specific complex can form (e.g., IKK). Because we estimated the number of trimeric TNFR1 receptors, we can calculate how many copies of a fully formed complex are available for trimeric receptor. We define here as copies required for isostoichiometry the number of copies of a given protein required to form at least one complex per trimeric receptor.

#### Copies recovered from AP-AQUA-MS.

Average ratio of endogenous and referenced spiked peptides provides the absolute amount of peptides and TNF-RSC members. Copy per cell of TNF-RSC members is estimated dividing the estimated absolute amount with the calculated number of processed cells in the AP-AQUA-MS experiment (2,400,000 or 2% of the starting material 1.2 × 10^8^ cells).

### Other Data Analyses.

#### Time course analysis.

Time course analysis was carried out using absolute amounts of TNF-RSC members as measured in the AP-AQUA-MS experiment, with the exception of UBASH3B, for which only relative intensities were used. Briefly, abundance of each analyte was divided by the receptor and normalized by the maximum value. Analysis was carried out in R.

#### Literature mining and data extraction.

Publications reporting information and data about PPI, stoichiometries, affinities, complexes isoforms, and other properties were systematically extracted from 1) Protein Data Bank (https://www.rcsb.org/), 2) BioGRID (https://thebiogrid.org/), and 3) the comprehensive resource of mammalian protein complexes (CORUM; mips.helmholtz-muenchen.de/corum/) and using targeted searches on 4) PubMed, 5) QInsight, and 6) generic search engines, as well as 7) references in publications. Data were collected until January 2020. We encountered several instances where we would have deemed a clear interpretation of the results problematic or impossible. Cases where the quality of the data was arguably highly suboptimal were discarded. In ambiguous cases, we attempted to adhere to the interpretation provided by the authors, based on the assumption that additional contextual information not presented in the publication could have supported it. For the sake of clarity, direct citations from the publications are reported in Dataset S10, whenever applicable and possible. Any additional information/interpretation that is not directly mentioned in the publication is reported in square brackets. Accuracy of curation was independently verified by R.C. and F.U.

#### Network visualization and GO analysis.

PPI networks in Fig. 2*G* and *SI Appendix*, Fig. S2*E*, were generated using Cytoscape (v3.6.0) (44).

## Supplementary Material

Supplementary File

Supplementary File

Supplementary File

Supplementary File

Supplementary File

Supplementary File

Supplementary File

Supplementary File

Supplementary File

Supplementary File

Supplementary File

Supplementary File

Supplementary File

Supplementary File

## Data Availability

Raw MS files, parameter files, Skyline sessions, and quantification files have been deposited for all acquired datasets in the ProteomeXchange Consortium via the Proteomics Identification Database (PRIDE): PXD019837, (45); project name: Identification of TNF-RSC (TNF receptor signaling complex) members in DDA], PXD019877, (46); project name: Identification of TNF-RSC (TNF receptor signaling complex) members in DIA], PXD019847, (47); project name: Blue Native Page based separation of the TNF-RSC), PXD019879, (48); project name: Absolute quantification of TNF-RSC members from isolated TNF-RSC from A549 cells), PXD019959, (49); project name: Absolute quantification and copy number estimation of the TNF-RSC members in A549 cells), PXD019878, (50); project name: Analysis of UBASH3B interactome in A549 and in HEK293 Flpin cell lines), PXD019903, (51); project name: Analysis of UBASH3B interactome in A549 and in HEK293 Flpin cell lines), and PXD031520, (52); project name: LUBAC interactors identification). Additional data and scripts are available from the corresponding authors on request. Previously published data were used for this work (14, 18, 34).
