# Supplementary material for: Novel biochemical, structural, and systems insights into inflammatory signaling revealed by contextual interaction proteomics
Source: Proc Natl Acad Sci U S A. 2022 Sep 30;119(40):e2117175119. doi: 10.1073/pnas.2117175119 (PMC9546619; doi:10.1073/pnas.2117175119)
Supplement: Supplementary File [file pnas.2117175119.sapp.pdf]

PNAS

# Supporting Information for
## Novel biochemical, structural and systems insights into inflammatory signaling revealed by contextual interaction proteomics

Rodolfo Ciuffa[1,†,*], Federico Uliana[1,†], Jonathan Mannion[3,†] ,Martin Mehnert[1], Tencho Tenev[3], Cathy Marulli[1], Ari Satanowski[2], Lena Maria Leone Keller[4], Pilar Natalia Rodilla Ramírez[1], , Pascal Meier[3], Alessandro Ori[5], Matthias Gstaiger[1], Pascal Meier[3,*], ,Ruedi Aebersold[1,6]*

**Affiliations:**
1 Institute of Molecular Systems Biology, ETH Zurich, 8093 Zurich, Switzerland
2 Max Planck Institute of Molecular Plant Physiology, Potsdam, Germany
3 The Breast Cancer Now Toby Robins Research Centre, The Institute of Cancer Research, London, UK
4 Institute of Molecular Biology and Biophysics, ETH Zurich, 8093 Zurich, Switzerland
5 Leibniz Institute on Aging, Fritz Lipmann Institute (FLI), Beutenbergstrasse 11, 07745 Jena, Germany
6 Faculty of Science, University of Zurich, Zurich, Switzerland
[†]These authors contributed equally
*Correspondence to: rodolfo.ciuffa2@gmail.com (R.C.), pascal.meier@icr.ac.uk (P.M.) aebersold@imsb.biol.ethz.ch (R.A)

**This PDF file includes:**

Extended Materials and Methods
Supplementary text
Figures S1 to S14
Legends for Dataset 1, 2, 3, 4, 5, 6, 7, 8, 9, 10, 11,12, 13
SI References

## Extended Materials and Methods

### Mass spectrometry data acquisition

**DDA data acquisition TNF-RSC AP-MS.** LC-MS/MS analysis was carried out on an Orbitrap Lumos mass spectrometer (Thermo Fisher) coupled to an EasynLC1200 (Thermo Scientific). Peptides were separated on a Acclaim PepMap 100 C18 (25 cm length; 75 μM diameter) using a 60 minutes gradient from acetonitrile 5 % to 37 % at a flow rate of 300 nL/min. The MS was operated in a data-dependent acquisition mode with the following parameters: one full FTMS scan in the *m/*z range 350-1500 with resolution of 120'000 and maximum injection of 100 ms; precursor ions with charge 2-5 were isolated with a window of 1.2 *m/z* and fragmented using HCD with a normalized collision energy of 27 %; and analyzed in FTMS at 30'000 resolution and a maximum injection time of 50 ms. The AGC target was set to 80'000, the cycle time to 3 seconds, and dynamic exclusion of 30 seconds.

**DDA data acquisition TNF-RSC in KO background.** LC-MS/MS analysis was carried out on a QExactive HF Hybrid Quadrupole-Orbitrap mass spectrometer (Thermo Fisher) coupled to an Acquity UPLC M-Class System (Waters). Peptides were separated on a Acquity UPLC M-Class column (15 cm length; 75 μM diameter) with a 120 minutes gradient from acetonitrile 5% to 37% at a flow rate of 300 nL/min. The MS was operated in a data-dependent acquisition mode with the following parameters: one full FTMS scan in the *m/z* range 350-1500 with resolution of 120000 and maximum injection of 50 ms; precursor ions with charge 2-7 were isolated with a window of 1.2 *m/z* and fragmented using HCD with a normalized collision energy of 28 %; and analyzed in FTMS at 30000 resolution with a maximum injection time of 50 ms. The AGC target was set to 100'000, and dynamic exclusion to 30 seconds.

**DDA data acquisition of UBASH3B AP-MS.** LC-MS/MS analysis of UBASH3B interactome was carried out on an Orbitrap Fusion mass spectrometer (Thermo Fisher) coupled to an Acquity UPLC M-Class System (Waters). Peptides were loaded on a commercial Symmetry C18 Trap Column (100Å, 5µm, 180µm*20mm, Waters) and separated on a HSS T3 Column (100Å,1.8µm, 75µm*250mm, Waters) using a 90 minutes gradient from acetonitrile 5% to 37% at a flow rate of 300 nL/min. The MS was operated in a data-dependent acquisition mode with the following parameters: one full FTMS scan in the *m/*z range 300-1500 with resolution of 120'000 and maximum injection of 50 ms; precursor ions with charge 2-7 were isolated with a window of 1.6 *m/z* and fragmented using HCD with a normalized collision energy of 30 %; and analyzed in IT (resolution 30'000 maximum injection time of 80 ms), cycle time to 3 seconds and dynamic exclusion of 25 seconds.

**DDA data acquisition of LUBAC AP-MS.** LC-MS/MS analysis of LUBAC interactome was carried out on an Orbitrap Q Exactive HF mass spectrometer (Thermo Fisher) coupled to an Acquity UPLC M-Class System (Waters). Peptides were loaded on a commercial Symmetry C18 Trap Column (100Å, 5µm, 180µm*20mm, Waters) and separated on a HSS T3 Column (100Å,1.8µm, 75µm*250mm, Waters) using a 90 minutes gradient from acetonitrile 5% to 40% at a flow rate of 300 nL/min. The MS was operated in a data-dependent acquisition mode with the following parameters: one full FTMS scan in the m/z range 300-1500 (resolution of 120'000 and maximum injection of 50 ms) and twelve MS2 scans (resolution 30'000 and maximum injection of 50 ms). Precursor ions with charge 2-7 were isolated with a window of 1.2 m/z and fragmented using HCD with a normalized collision energy of 28 %. A dynamic exclusion of 30s was applied.

**DIA data acquisition.** LC-MS/MS analysis was carried out on an Orbitrap Lumos mass spectrometer (Thermo Fisher) coupled to an EasynLC1200 (Thermo Scientific). Peptides were separated on a Acclaim PepMap 100 C18 column (25 cm length; 75 μM diameter) with a 120 minutes gradient from acetonitrile 5% to 37% at a flow rate of 300 nL/min. The MS was operated in a data-independent acquisition mode with the following parameters: one full FTMS scan in the *m/*z range 350-1500 with resolution of 120'000 and maximum injection of 100 ms with an AGC target of 200'000; followed by 40 fixed windows from 399.5 to 1000.5 *m/z* with 1 *m/z* overlap at 30000 resolution with an injection time of 50 ms and an AGC of 500'000 for a cycle time of 3 seconds. Precursor ions were fragmented with HCD, normalized collision energy of 27%.

**PRM data acquisition TNF-RSC AP-MS.** Targeted analysis for the AQUA experiments were carried out on a QExactive HF Hybrid Quadrupole-Orbitrap mass spectrometer (Thermo Fisher) coupled to an Acquity UPLC M-Class System (Waters). Peptides were loaded on a commercial Symmetry C18 Trap Column (100Å, 5µm, 180µm*20mm, Waters) and separated on a HSS T3 Column (100Å,1.8µm, 75µm*250mm, Waters) using a 90 minutes gradient from acetonitrile 5% to 37% at a flow rate of 300 nL/min. Isolation window was set at 1.2 m/z. Peptides were fragmented with a collision energy at 28% (HCD) and detected in an Orbitrap. Affinity purified samples were injected three times with two different methods depending on the intensity of the monitored peptides, with Orbitrap fill time and resolution set at 54/118 ms and 30'000/60'000, respectively.
**PRM data acquisition TNF-RSC lysate.** Targeted analysis for the AQUA experiments were carried out on a QExactive HF Hybrid Quadrupole-Orbitrap mass spectrometer (Thermo Fisher) coupled to an Acquity UPLC M-Class System (Waters). Peptides were loaded on a commercial Symmetry C18 Trap Column (100Å, 5µm, 180µm*20mm, Waters) and separated on a HSS T3 Column (100Å,1.8µm, 75µm*250mm, Waters) using a 90 minutes gradient from acetronitrile 5% to 37% at a flow rate of 300 nL/min. Isolation window was set to 1.2 m/z. Peptides were fragmented with a collision energy at 28% (HCD) and detected in an Orbitrap. Lysates were injected three times with three different methods depending on the intensity of the monitored peptides, with Orbitrap fill time and resolution set at 54/118/236 ms and 30'000/60'000/120'000, respectively.
**PRM data acquisition of AP-BNPAGE-MS.** Targeted data acquisition (PRM) for the AP-BNPAGE-MS experiments were carried out on an Orbitrap Lumos mass spectrometer (Thermo Fisher) coupled to EasynLC1200 (Thermo Scientific). Peptides were separated on a Acclaim PepMap 100 C18 (25 cm length; 75 µM diameter) using a 40 minutes gradient from acetonitrile 5 % to 37 % at a flow rate of 300 nL/min. Signal generated from known amounts of the corresponding heavy peptides was measured every 5 runs. Isolation window was set to 1.4 m/z. peptides were fragmented with a collision energy of 30% (HCD) and detected in an Orbitrap using a fill time of 256 ms and a resolution of 120'000 and 246ms IT, with the exception of the iRT peptides, whose spectra were recorded with a resolution of 15'000 and 22ms IT.
**PRM data acquisition UBASH3B IP-MS**. Targeted data acquisition for the UBASH3B IP-MS were carried out on an Orbitrap Lumos mass spectrometer (Thermo Fisher) coupled to EasynLC1200 (Thermo Scientific). Peptides were separated on a Acclaim PepMap 100 C18 (25 cm length; 75 µM diameter) using a 90 minutes gradient from acetonitrile 5% to 37% at a flow rate of 300 nL/min. Isolation window was set to 1.4 m/z. Peptides were fragmented with a collision energy at 30% (HCD) and detected in an Orbitrap, using 118 ms fill time and 60000 resolution.

**Mass Spectrometry Data Analysis**
**DDA.** MS1 intensities from shotgun data were analyzed using MaxQuant (v 1.5.2.8) (*1*). UniProtKB/Swiss-Prot database (download 2019.06.15, plus GST-TNFα sequence) using the following parameters: fully tryptic peptides; fixed modifications: Carbamidomethyl; variables modifications: acetylation, oxidation (M), phosphorylation (STY); MS and MS/MS mass tolerance set to 20ppm; FDR <1% at the protein level; LFQ with a minimum of 2 unique peptides; match-between-runs and iBAQ options. For the identification of UBASH3B interactors Uniprot/Swiss-Prot database (download 2020.04.01) and default parameters were used with match between runs and LFQ intensity based on top 2 unique peptides. Customized R scripts were used to carry out the statistical analysis; steps included median or bait-normalization, imputation of missing values using sampling from the 5 lowest quantiles, calculation of fold changes and associated non-adjusted and adjusted (BH) p-values.

**DIA.** Spectral library was generated with Spectronaut 13 (v.13.2.190709, Biognosys) using a hybrid library combining results from MaxQuant analysis and Pulsar extraction. Settings for library query were as follows: tolerance of 10 ppm for precursors, 25 ppm for fragment ions and a dynamic rT extraction window with non-linear iRT. Precursors and proteins were identified with a q-value cutoff of 0.01. Data normalization (TIS) and filtering were performed using MapDIA (Teo et al., 2015), where a standard deviation factor of 2 and a minimal correlation of 0.2 were used to filter robust fragment ions with a minimum intensity of 500. Filtering strategy at protein level included following conditions: transitions required to be detected in at least 1 of 3 replicates; median normalization of transition levels; peptide values resulting from the sum of 3-5 top transitions, and protein values

resulting from the sum of 1-3 top peptides. Missing values were imputed using a randomized distribution of values from the 5 lowest percentiles.

**PRM.** Representative peptides for the ~30 TNF-RSC proteins were selected based on the following criteria: proteotypic; few or no known posttranslational modifications; tryptic; good response factor and linearity, as measured by targeted/untargeted MS, and/or reported in peptide atlas (http://www.peptideatlas.org/). AQUA peptides used in this study were purchased from Thermo Fisher Scientific (Aqua QuantPro). All targeted data were analyzed and quantified using Skyline (version: daily(beta)) (*2*). Generally, manual peak-picking from PRM data was carried out using the following criteria: *m/z*>200; ppm<5; of multiple available charged states, the one with the best features was chosen; identification was based on co-elution of light and heavy peptides and matching peak shape. For all analyses, peak-picking and data evaluation was carried out independently by RC and FU and results compared for agreement. Quantitative peaks results obtained from Skyline analysis (version: daily(beta)) were exported and downstream analyses carried out in R using customized scripts. For AP-BNPAGE-MS dataset, downstream data analysis was performed in R, and included: (i) standardization; (ii) imputation of missing values (lowest value from the entire data matrix); (iii) detection and correction of outlier measurements, where outliers were defined as data points whose intensities were at least 5-fold lower than the adjacent data points, with the average of which they were replaced; (iv) curve smoothening, using rollmean function in the R package zoo. Because the signal in the first fractions is up to 250 fold (-DUB) and 35 fold (+DUB) higher than in subsequent fractions (Figure 3D/E), we have chosen fraction 3 and fraction 5 as reference normalization fractions (for all proteins) for +DUB and -DUB samples, respectively; while we have used a different color range to code for the intensities of the first fractions.

## Supplementary Information Text

**Mid-confidence interactors identifications.** Identified proteins with at least 1 log2 FC were scored by the following criteria: (1) Predicted protein complexes, as reported in (*3*); (2/3) number of reported PPIs and evidences supporting them, based on BioGRID; (4) Cocitation, as determined using the R package RISmed. Unsupervised hierarchial clustering (Fig. S2C) was carried out in R. By this means, we could identify a clear high scoring cluster containing AZI2 (also known as NAP1), TAX1BP1 and cdc37. The former has been reported to mediate the recruitment of the kinase TBK1 to the TNF-RSC (*4*); TAX1BP1 is one of its direct interactors, previously shown to regulate the termination of TNFα signaling (*5*); while cdc37 is an HSP90 cochaperone and thus a well characterized interactor of the IKK complex (*6*). We next focused on proteins that are selectively enriched against the His-tagged TNFα control, i.e. putative stimulus-independent binders of the receptor. Interestingly, we found three closely related proteins belonging to the KCTD family, which are best known as substrate adapters for Cullin-RING E3 ligases (*7*). Intriguingly, KCTD proteins have been reported to interact with CYLD, TNFα, TNIP2 and OPTN (BioGRID v.3.5; Fig. S2E, only genes associated with GO “Defense response” are shown), suggesting a potential role in innate immune signaling. Furthermore, a recent screen indeed identified KCTD5 as a putative negative regulator of p65 (*8*). Conversely, we also examined those interactors that are specifically enriched in the unstimulated control vs the His-tagged TNFα control. As expected, we found an enrichment in membrane proteins, and found that the large majority of them is associated with lipid rafts (*9*), consistent with a role for lipid rafts in the activation of TNFα signaling (*10*) (not shown). Finally, the confident identification of TRAF6 and TRAF3 as proteins associated to the TNF-RSC (Fig. 2B), not discussed in the main text, is similarly noteworthy, since the latter is traditionally associated with the activation of the non-canonical NF-κB signaling pathway and only sparse evidence links the former to TNFα signaling.

**Detailed interpretation of the AP-BNPAGE-MS experiment results.** We interpret the regular pattern observed in the AP-BNPAGE-MS +DUB as resulting primarily from two processes: (i) the removal of heterogenous ubiquitin chains, resulting in a reduction in protein isoforms and sharpening of migration peaks. This is particularly evident, on a WB level, for RIPK1 (Fig. S8B),

and on the BNPAGE, for the TNFR1 (Fig. 3A); (ii) more importantly, the disassembly of the signalosome in discrete, partially overlapping fragments. The comparison of the ratios of proteins belonging to the same complex across peaks (Fig. S10B/C), which are roughly constant, also rules out that peaks are formed by different isoforms of the same complex (i.e. with different stoichiometries). Finally, several lines of evidence, including stability of reference peptides (iRT peptides and TNF-RSC heavy peptides), stability of MS1 total ion current, and consistent pairing of reference heavy and light peptides retention time, indicate that technical factors do not contribute to the generation of this periodic pattern (Fig. S9A/C). Taken together, these observations suggest that the observed signal distribution is the result of the progressive disassembly – driven by the removal of discrete, ubiquitin-bound subunits – of the signalosome, where peaks are generated by different combinations of stoichiometrically stable complexes in the high MW region of the gel, and that individual complexes would further disassemble in the low MW range. Because of the proximity to the limit of detection and the complex pattern of disassembly, it is difficult to draw conclusions about the behavior of specific proteins, even though some stand out as particularly noteworthy. UBASH3B, for instance, does not clearly co-migrate with any of the TNF-RSC members that, based on the affinity purification experiments presented in Fig 2F/G and S4A/C, are most strongly associated with the phosphatase. This may suggest that ubiquitin chains are mediating its recruitment, as they are disassembled in our experiment.

**Detailed interpretation of the stoichiometry of the TNF-RSC complexes. Absolute stoichiometry of the core complex.** Of the stoichiometries determined for the core complexes, BIRC2 is found at an average ~1:4/5 ratio with TRAF2, compared to a 1:3 ratio observed *in vitro* (*9*). Variation from the 1:3 value could be explained by (i) the confounding additional presence of TRAF1, which is known to form heterotrimeric complexes with TRAF2, and BIRC3; (ii) the known association of TRAF2 with the TBK1 complex; (iii) loss of a portion of BIRC2 during the purification; (iv) slightly higher abundance of TRAF2 as compared to the receptor. While 1:3 arrangement reflects available structural data, it is known that BIRC2 activity relies on its dimerization. Several publications have previously suggested that the receptor, TRAF6 or NEMO could mediate the cross-linking of different signalosomes in a large lattices (*11,12,13,14*). This is compatible with the idea that two BIRC2 molecules would dimerize from two different signalosomes and by this means represent an alternative mode of cross-linking, as previously suggested (*15*). **Stoichiometry of the IKK Complex (IKKA, IKKB, NEMO).** We found that the IKK complex is assembled in either one of the following stoichiometries: $4_{NEMO}:1_{IKKB}:1_{IKKA}$ (iBAQ) or a $\sim 9_{NEMO}:3_{IKKB}:1_{IKKA}$(AQUA) stoichiometry. We observe here that several biochemical studies have suggested that the IKK complex contains an equimolar amount of NEMO and (IKKA/B; Dataset 10). Our data indicates otherwise. We found approximately twice as many molecules of Nemo as molecules of the catalytic subunits, suggesting that the IKK complex composition at the receptor differs from its average cytoplasmic composition. Because we found that NEMO is generally never in excess with respect to the catalytic subunits in other MS data (*16*)(Fig. S14A; FigS14B), we rule out that our results depend on a MS-specific bias towards NEMO. These lines of evidence establish an asymmetry between cytoplasmic and receptor-bound composition of the IKK complex, which can be described as follows: $\sim(2_{NEMO}:2_{IKKA/B})_{cytoplasm} \rightarrow \sim 2_{NEMO}+(2_{NEMO}:2_{IKKA/B})_{receptor}$. Second, we asked what is the most likely absolute stoichiometry of the complex, the two biochemically most plausible solutions being $2_{NEMO}+(2_{NEMO}:2_{IKKA/B})$ and $4_{NEMO}+(4_{NEMO}:4_{IKKA/B})$. Due to the elongated shape of NEMO, MW estimates based on SEC are unfortunately unreliable. On the other hand, glycerol gradient experiments have produced estimates of ~350-450 kDa for the complex, while the lowest IKK complex peak detectable in our BNPAGE experiment in absence of DUB (Fig. 3D, IKKA signal) is located at about ~500 kDa. This evidence is compatible with a model of an IKK tetramer binding two NEMO dimers, whose number would double upon stimulation (Fig. 3H), but is not conclusive. Finally, we asked what is the relative contribution of IKKA/B to the complex. Here we observe that there are several different solutions that would explain the present data or most published data, and it is impossible to arrive at a unique solution without knowledge about IKK complex isoforms. Measurement of lysate protein abundance indicates IKKA/IKKB ratios similar to the ones estimated in our AP-MS by AQUA (Fig. S14E), suggesting an overrepresentation of IKKB in the complexes. At the same time, SEC-MS data indicates the existence of different complex isoforms (Fig. S12H).

## Supplementary Figures legends

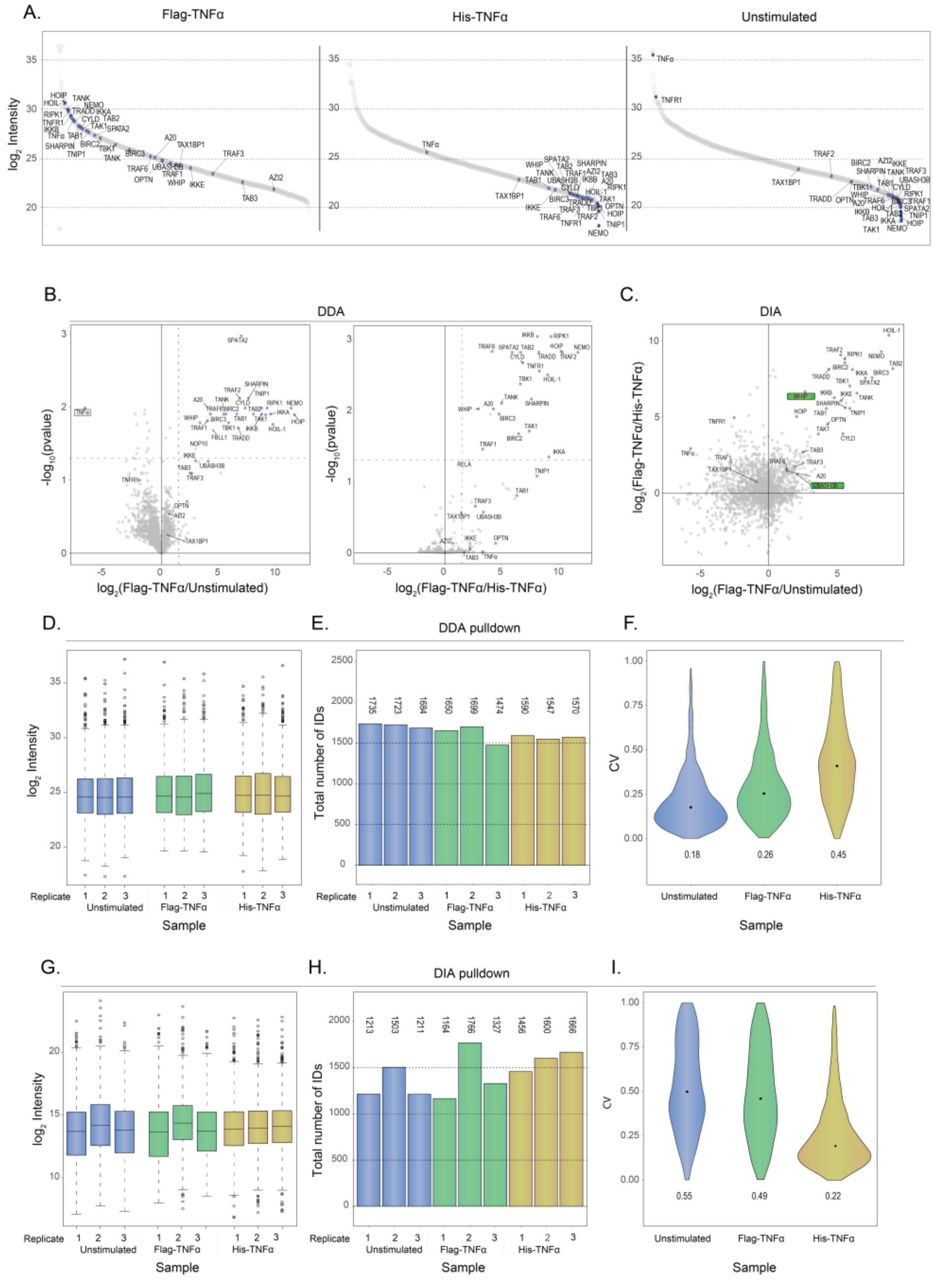


**Figure S1. Quality controls on affinity-purified TNF-RSC analyzed by DDA and DIA (A)** Intensity distribution ($log_2$) of TNF-RSC members across treated and control samples acquired by DDA. (**B**) Volcano plots of individual controls (Unstimulated and his-TNFα, DDA data). (**C**) Scatterplot of $log_2$FC values showing protein enrichment across two controls (DIA data; analogous to Fig. 2B). (**D/G)** Boxplot of $log_2$ intensities distribution across samples (DDA and DIA data, respectively). (**E/H)** Number of protein IDs identified in individual replicates (DDA and DIA data, respectively). (**F/I)** Violin plot showing coefficient of variation values of raw data (DDA and DIA data, respectively).

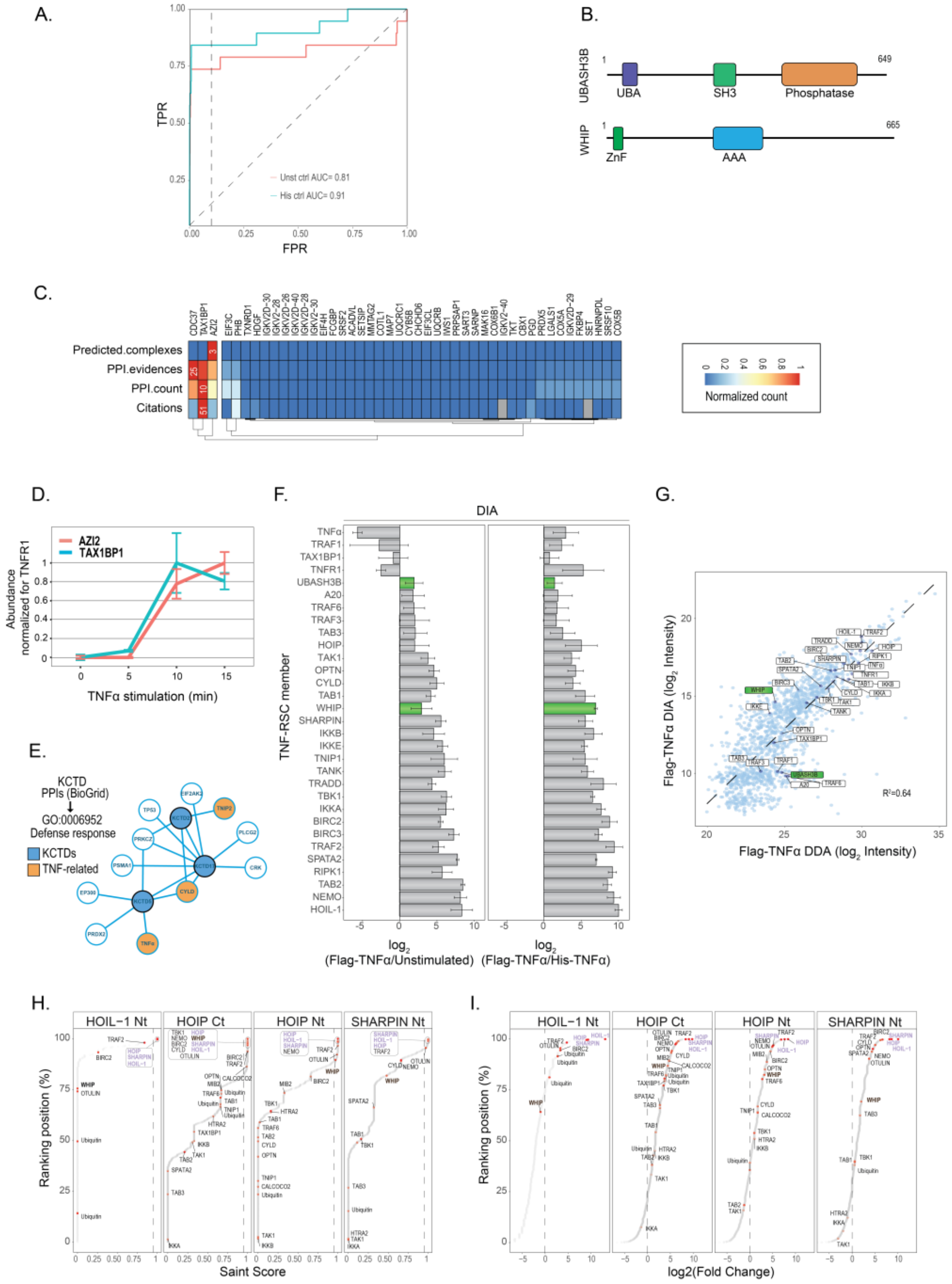

**Figure S2. Identification of mid-confidence interactors and further validation by DIA of UBASH3B and WHIP recruitment to the TNF-RSC. (A)** Receiver operating characteristic curves (ROC) for the endogenous pulldown. Interactors reported as interactors of TNFR1 in BioGRID are considered as true positive. The ROC evaluates two different approaches (His-TNFα control and unstimulated control) **(B)** Primary sequence of UBASH3B and WHIP. (**C)** Clustering of mid-confidence interactors by multiple criteria isolates three known TNF-RSC associated proteins. (**D)** The recruitment of AZI2 and TAX1BP1 to the TNF-RSC is confirmed by targeted proteomics on isolated signalosomes (A549 cells) across the indicated time points after stimulation; the data is based on the same experiment presented in Fig. 2C and S13A. (**E)** Previously reported interactions (BioGRID) of KCTD proteins indicate putative association with TNF-RSC members. (**F)** Log$_2$FC enrichment of signalosome proteins identified in the treated and control samples from the DIA dataset. WHIP and UBASH3B are highlighted in green. (**G)** Scatterplot indicates correlation of protein intensities (log$_2$) between DIA and DDA data. WHIP and UBASH3B are highlighted in green. (**H, I**) Identification of interactors for HOIL-1, HOIP and SHARPIN using Saint Score and enrichment of spectral counts versus three GFP control. All identified known members and associated component of TNF-RSC are highlighted in the plot.

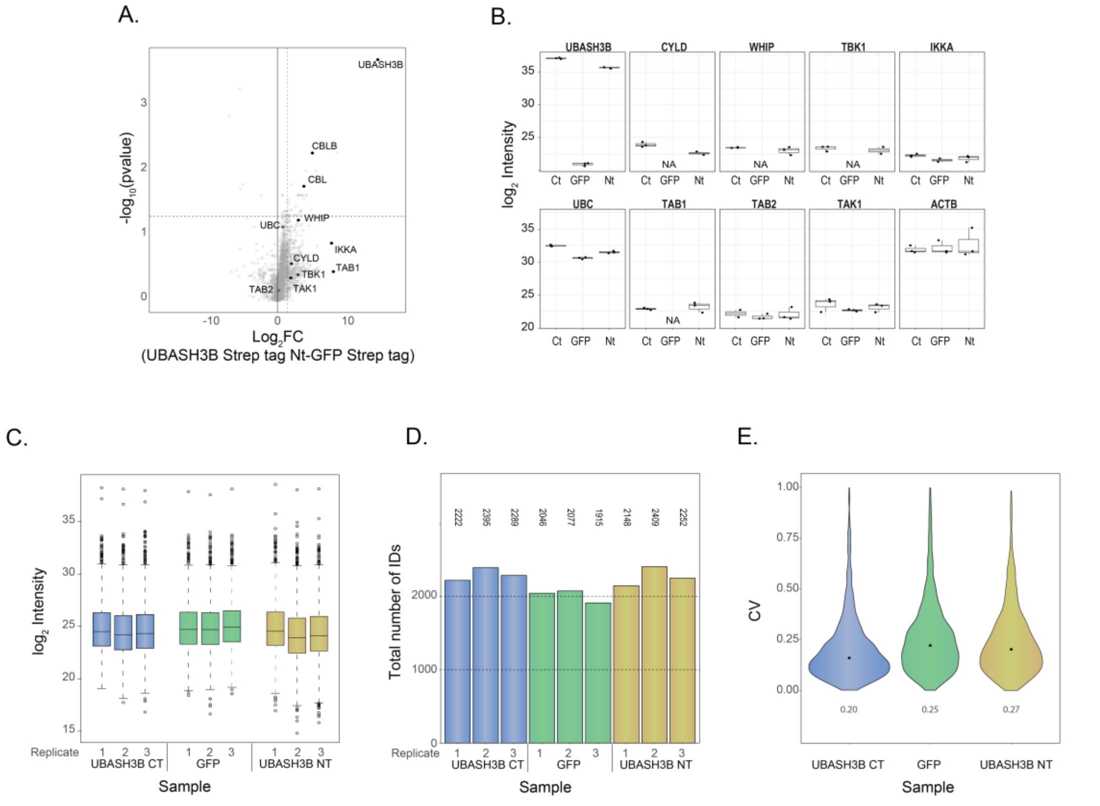


**Figure S3. Characterization of the interactome of ectopically expressed UBASH3B by AP-MS in HEK293 cells**. (**A**) Volcano plot of affinity purified N-terminally tagged UBASH3B against a GFP control indicates enrichment of TNF-RSC proteins. (**B)** Abundance of selected TNF-RSC proteins in AP-MS of C- and N-terminally tagged UBASH3B as well as GFP analyzed by DDA. Actin is shown as control. (**C)** Boxplot of $log_2$ intensities distribution across samples. (**D)** Number of protein IDs identified in individual replicates. (**F)** Violin plot showing coefficient of variation values of raw data.

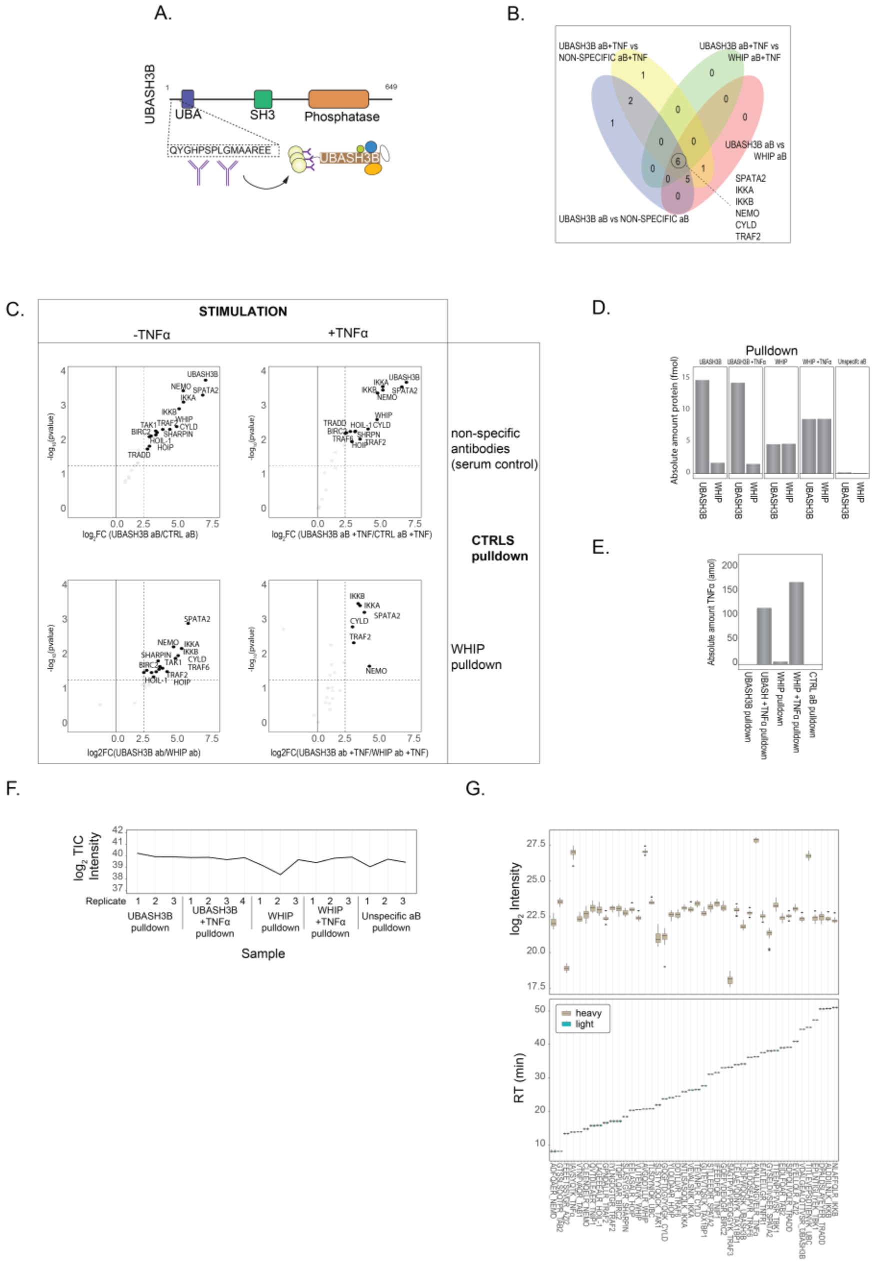

**Figure S4. Characterization of the UBASH3B interactome by IP-MS in A549 cells. (A)** Primary sequence of UBASH3B and peptide used to raise antibody for endogenous IP-MS. (**B)** Venn diagram with proteins identified as consistently significant across the four conditions shown in panel S3D. Four conditions correspond to UBASH3B IP-MS against IP-MS with non-specific antibodies (serum control) +/- ligand and WHIP IP-MS +/- ligand. The WHIP control was used as an additional, more stringent control. (**C)** Volcano plots of UBASH3B endogenous affinity purification against four controls, as described in panel C legend. UBASH3B IP-MS interactors were filtered against control purification with non-specific antibodies (serum control) in presence or absence of TNFα stimulation (upper panel, left and right respectively). Since UBASH3B and WHIP antibodies were raised together and purified sequentially, we added WHIP purification as a more stringent control to address concerns regarding potential antibodies cross-contamination. WHIP purification was used as control in presence and absence of TNFα stimulation (lower panel, left and right, respectively) (**D)** Average amount of WHIP in UBASH3B in pulldowns described in panel C. (**E**) Average amount of ligand in the indicated conditions. (**F)** Total ion current (TIC) from UBASH3B IP-MS targeted analysis. **(G)** Signal from reference heavy peptides (top) and consistent pairing of heavy and light peptides (bottom) measured in UBASH3B IP-MS sample.

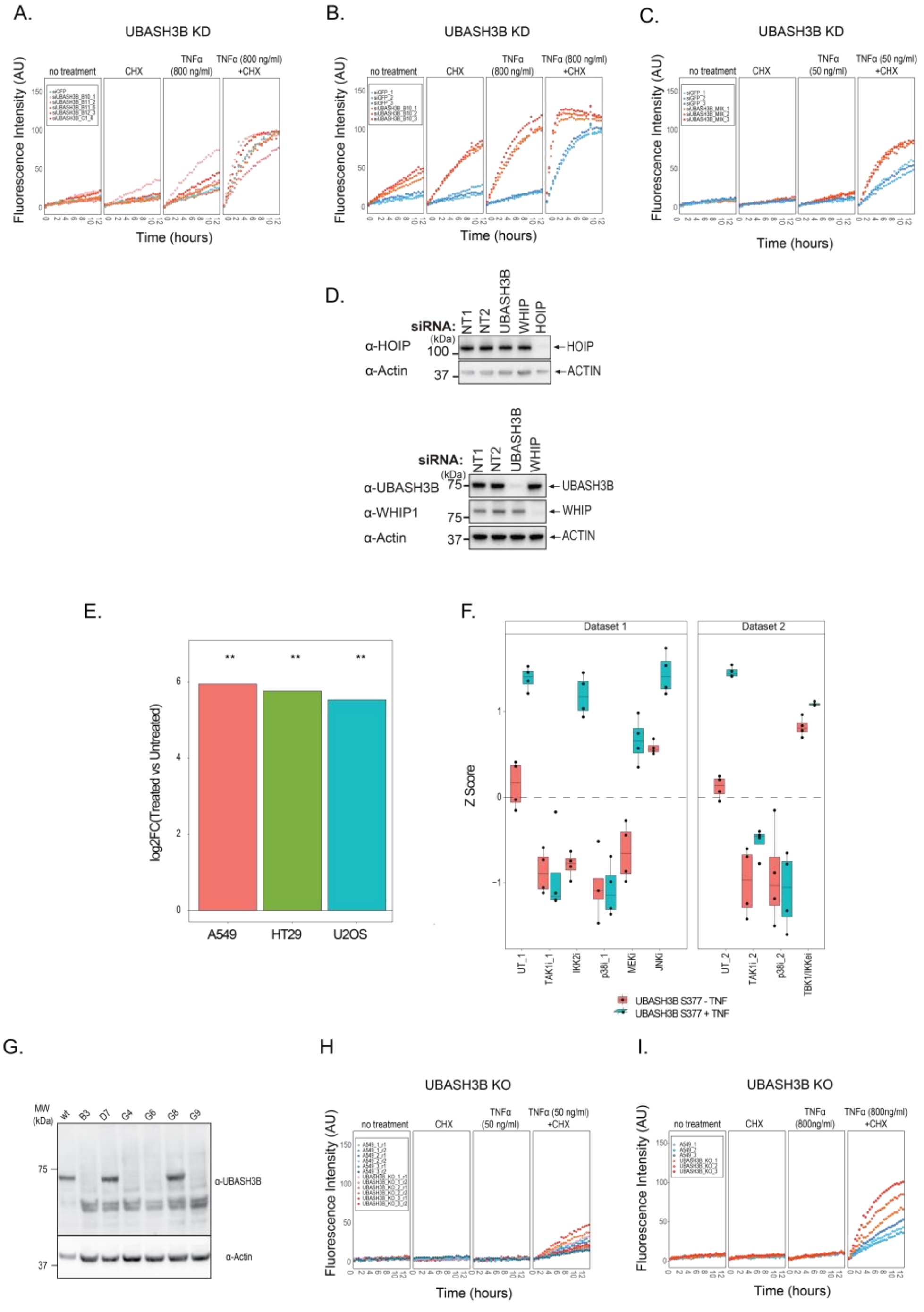


**Figure S5. Link between TNF signaling and UBASH3B. (A/B/C)** DEVDase assay with the indicated siRNAs and ligand concentrations in UBASH3B KD A549 cell line. (**D**) Validation of knockdown efficiency of UBASH3B, WHIP and HOIP by immunoblot. (**E**) UBASH3B S377 phosphorylation following TNFα treatment (15 minutes) in 3 human cell lines. Plotted data is from the screening data published by Tanzer and colleagues (*17*) and is extracted from data matrix in Supplementary Information 1. (**F**) UBASH3B S377 phosphorylation following TNFα treatment and addition of the indicated inhibitors. Plotted data is from the screening data published by Tanzer and colleagues (*17*) and is available at the following website: http://tnfviewer.biochem.mpg.de/TNFviewer_final. (**G**). Immunoblot against UBASH3B in clones of UBASH3B KO A549 cells. (**H/I**) DEVDase assay with different ligand concentrations in UBASH3B KO A549 cell line.

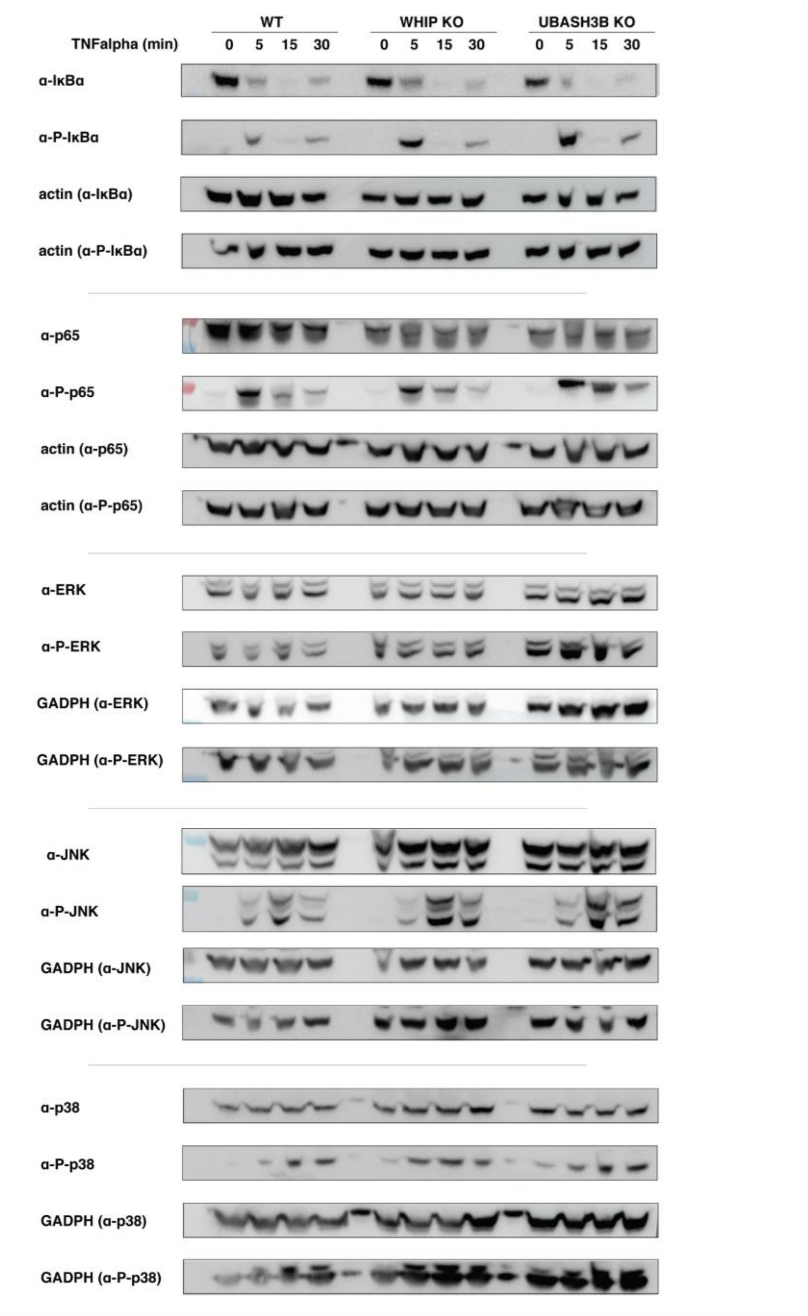


**Figure S6. Immunoblot analysis of signaling progression in UBASH3B KO A549 cell line.** Immunoblot against the indicated proteins in A549 WT/UBASH3B KO/WHIP KO across the indicated time points after TNFα stimulation.

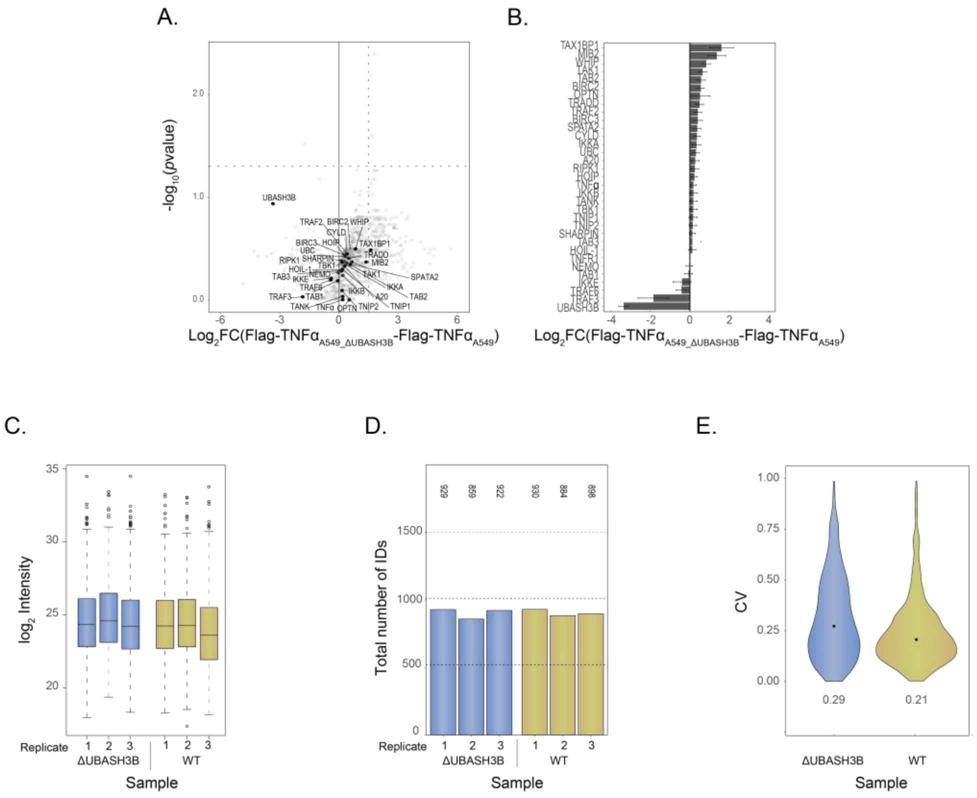


**Figure S7. Characterization of the TNF-RSC interactome in UBASH3B KO A549 cells.** (**A)** Volcano plot of TNF-RSC affinity purified with tagged TNFα in UBASH3B KO A549 cells against using WT A549 cells as a control. (**B**) $Log_2FC$ enrichment for selected TNF-RSC members. (**C)** Boxplot of $log_2$ intensities distribution across samples. (**D)** Number of protein IDs identified in individual replicates. (**E)** Violin plot showing coefficient of variation values of raw data.

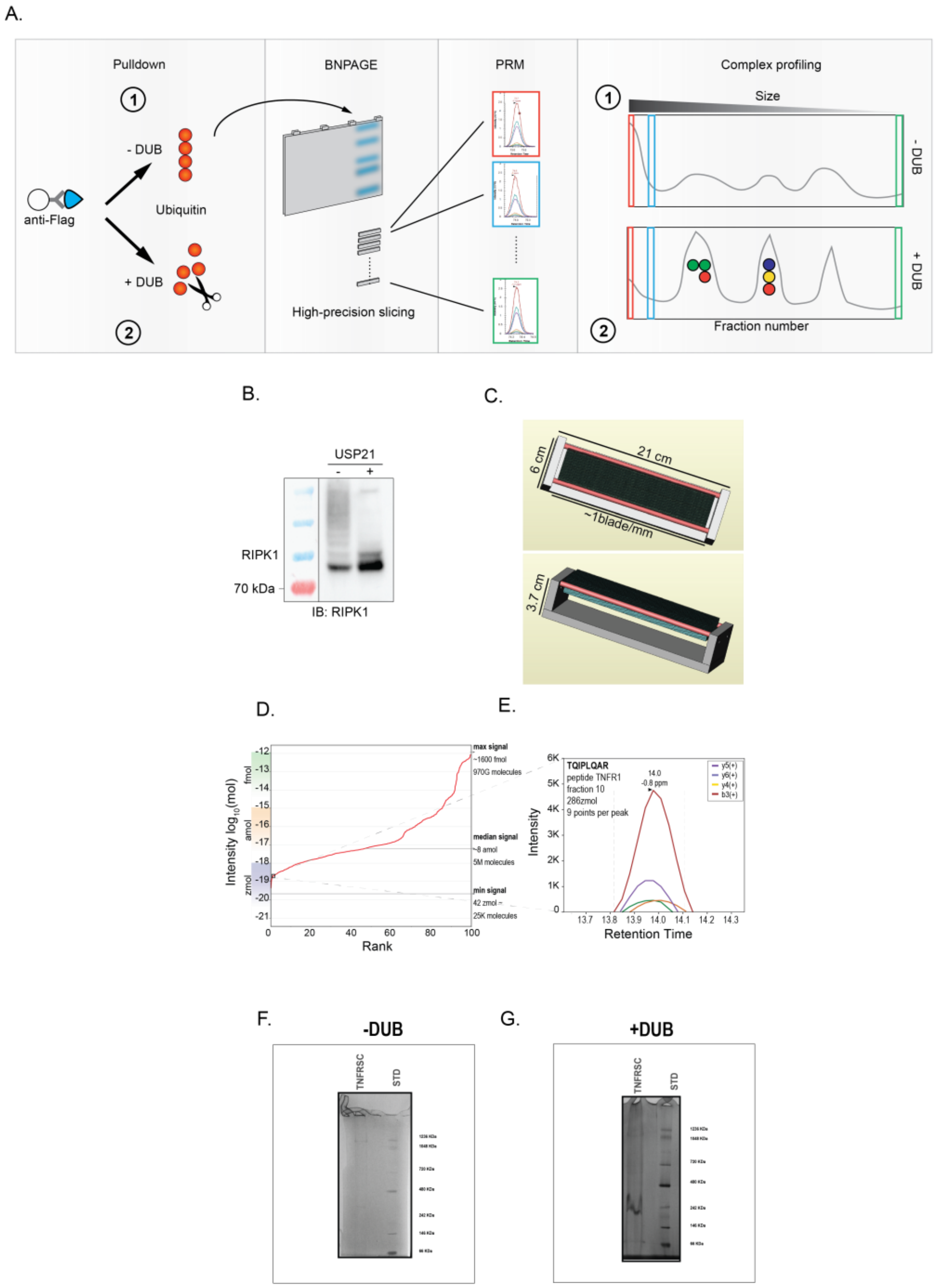


**Figure S8. Experimental design of the TNF-RSC fractionation by AP-BNPAGE-MS**. (**A**) Experiment design: affinity purified TNF-RSC with or without prior addition of the DUB USP21 were separated onto a BNPAGE gel. The gel was sliced with a custom-made gel slicer (panel **C**) and the extracted, digested proteins were analyzed by MS targeted proteomics. (**B**) Treatment of affinity purified TNF-RSC with USP21 induces a nearly quantitative reduction of ubiquitin chain signal as assessed by RIPK1 immunoblot. (**C**) Rendering of the high-precision gel slicer custom-designed for this study. (**D**) Distribution of peptide intensity and amount estimated from heavy AQUA standards (USP21-treated sample). While most signal falls within the attomole range, limit of detection of peptide is in the high zeptomole range. (**E)** Example of fragment ions peak group from a peptide with an estimated abundance in the zeptomole range where not only detection but also reliable quantification was possible. (**F/G**). Scan of gel image of BNPAGE gels in absence (left) and presence (right) of USP21.

A.

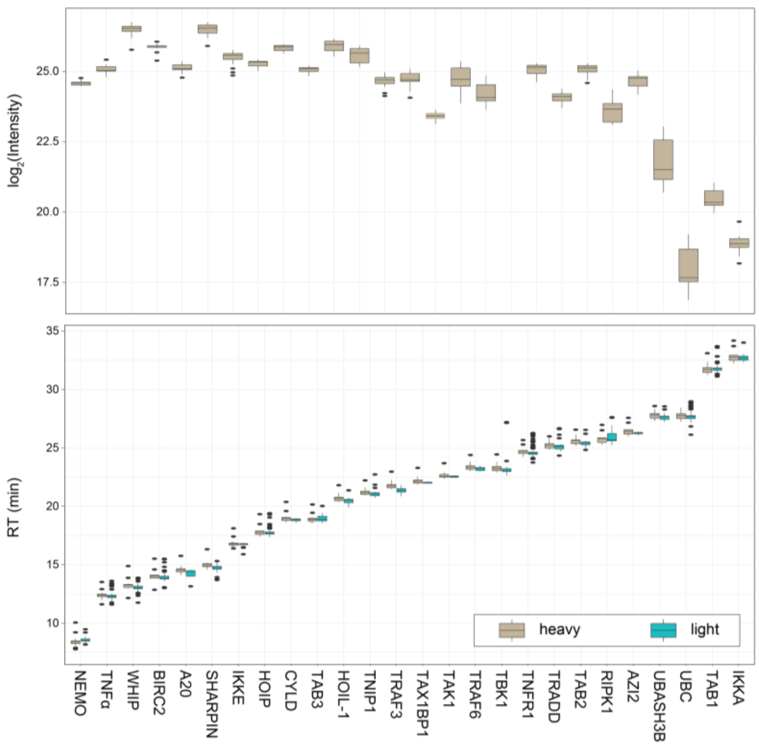


B.

| Gene name | Peptide NO DUB | Peptide PLUS DUB |
|---|---|---|
| IKKA | IIVHTVQSQDR | IQLPIIQLR |
| IKKB | ALDDILNLK | / |
| TAK1 | EIEVEEVVGR | EIEVEEVVGR |
| TNFα | IAVSYQTK | IAVSYQTK |
| UBC | TITLEVEPSDTIENVK | TITLEVEPSDTIENVK |
| TNFR1 | EATLELLGR | EATLELLGR |
| A20 | / | LDEANLPK |
| TRAF2 | QQEHEVQWLR | / |
| TRAF1 | / | LLAELEGK |
| TRAF3 | SAGTPVFVPEQGGYK | SAGTPVFVPEQGGYK |
| BIRC3 | ESNDLLLIR | / |
| BIRC2 | TQIPLQAR | TQIPLQAR |
| RIPK1 | LLGVIIEEGK | LLGVIIEEGK |
| IKKE | / | LVGEEGQSIYK |
| TNIP1 | VPEVVALGAAEK | IFEEDFQR |
| TRADD | SDPQLIVQLR | SDPQLIVQLR |
| TAB1 | VLLQAFDVVER | VLLQAFDVVER |
| TAX1BP1 | GLTEVTQSLK | LELAEVQDNYK |
| TAB3 | / | EVDLLQSR |
| HOIP | YLQLDAAR | YLQLDAAR |
| WHIP | / | ALAAEEIR |
| HOIL-1 | ALLTPEDYQR | ALLTPEDYQR |
| SHARPIN | GPPPEADLPR | GPPPEADLPR |
| AZI2 | EVELLQLR | EVELLQLR |
| CYLD | TEIVNPLR | TEIVNPLR |
| TAB2 | EIDLFQAR | EIDLFQAR |
| TBK1 | LFAIEEETTTR | LFAIEEETTTR |
| SPATA2 | GYSELDIVSER | / |
| TRAF6 | LTILDQSEAPVR | LTILDQSEAPVR |
| NEMO | AQVTSLLGELQESQSR | ADFQAER |
| TANK | / | / |
| UBASH3B | / | VDALGEALQTTVSR |

C.

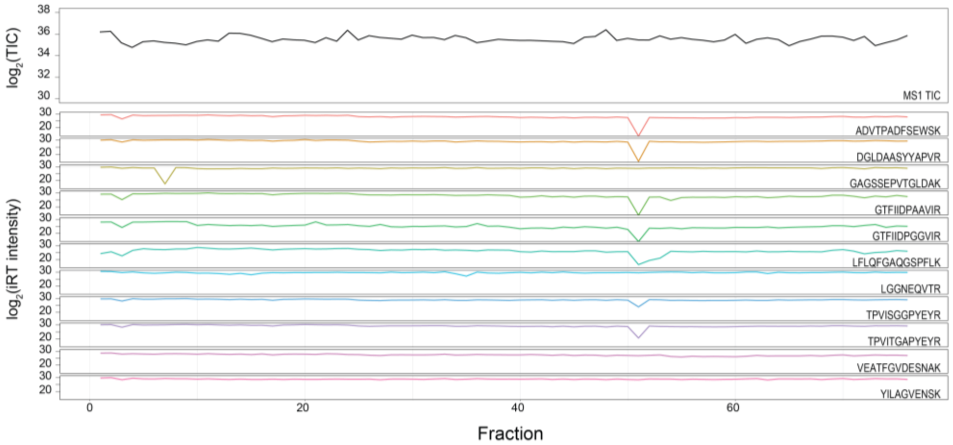


**Figure S9. Quality controls for AP-BNPAGE-MS experiment. (A)** Signal intensity from reference heavy peptides (top) and consistent RT pairing of heavy and light peptides (bottom) measured in BNPAGE sample (treated with DUB USP21). (**B)** List of peptides monitored by PRM in the AP-BNPAGE-MS experiments. (**C)** Total ion current (TIC) profile (top) and signal intensity of iRT peptides (bottom) from the AP-BNPAGE-MS sample treated with DUB USP21.

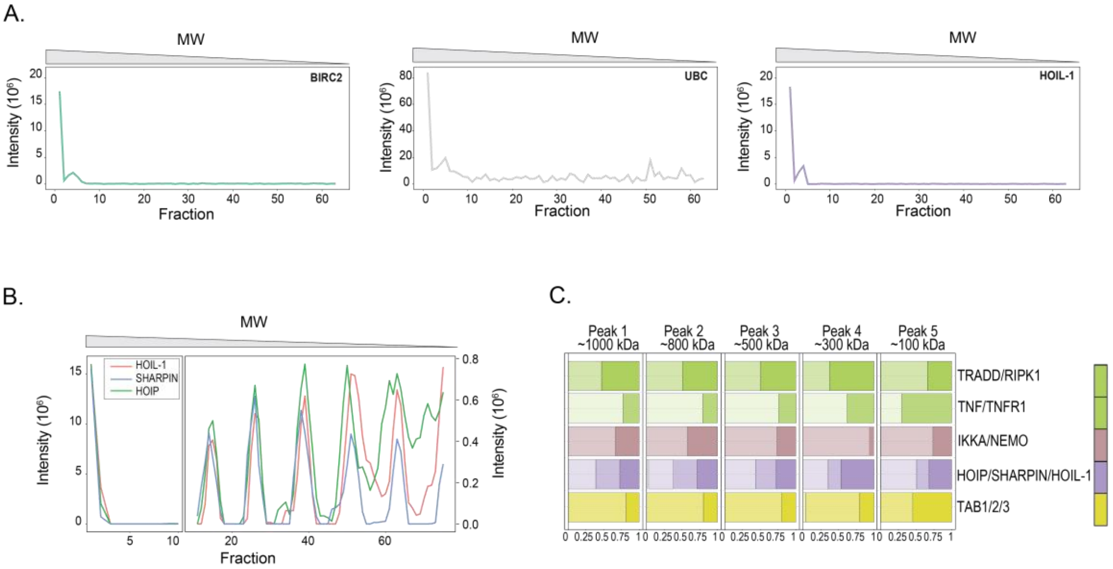


**Figure S10. AP-BNPAGE-MS profiles of TNF-RSC untreated or treated with DUB.** (**A)** Signal distribution for selected proteins (BIRC2, UBC and HOIL-1) reveals protein accumulation in the stacking region of the gel. (**B)** AP-BNPAGE-MS migration profiles of LUBAC members after treatment with DUB USP21. (**C)** Relative abundance of membrane proximal proteins (green), IKK (brown), LUBAC (purple) and TAB/TAK complex members (yellow) remain constant across the first three peaks of the BNPAGE after treatment with DUB USP21.

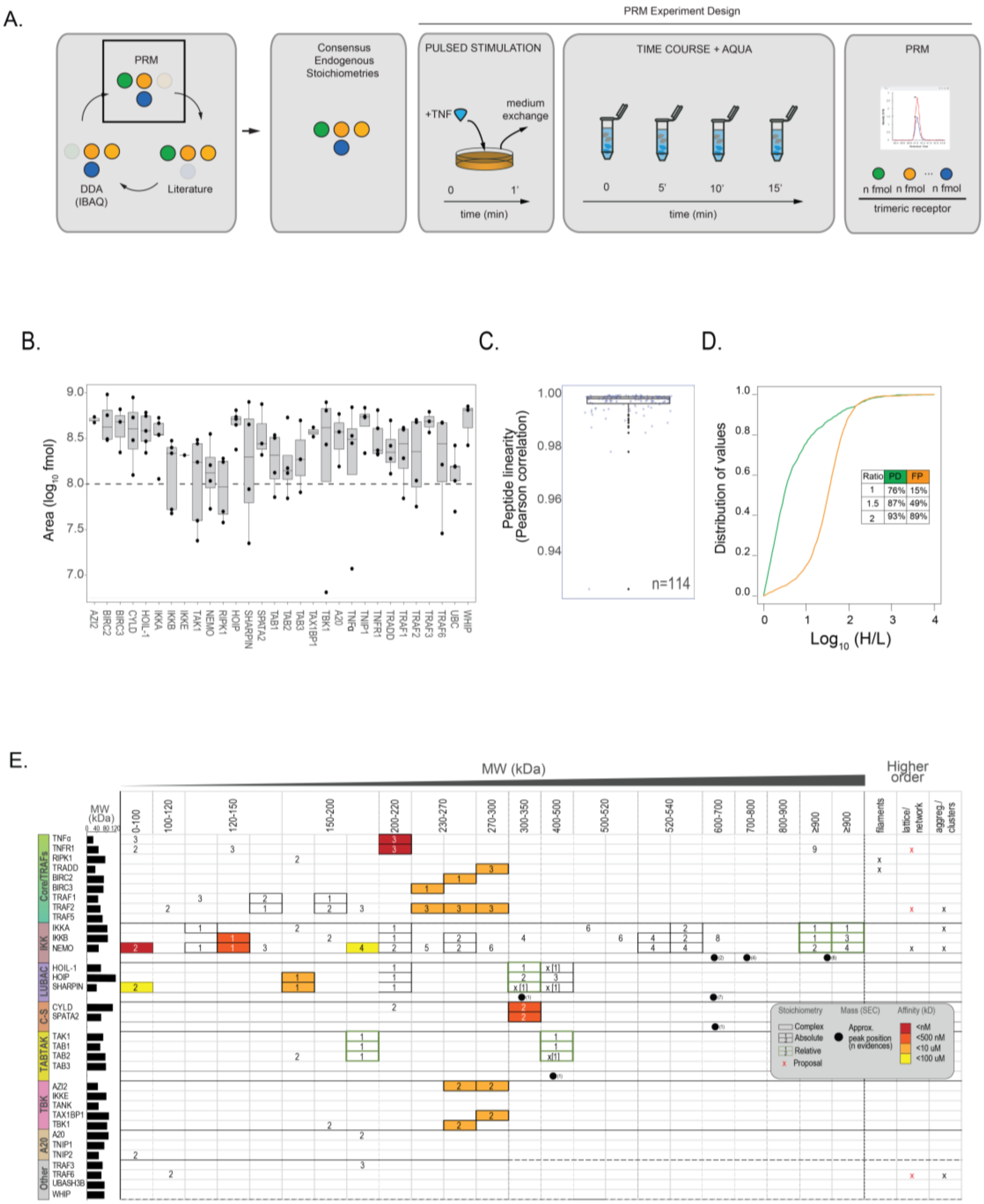


**Figure S11. Assessment of TNF-RSC members stoichiometry by orthogonal approaches (DDA, AQUA and literature curation). (A)** Design (left): Stoichiometries obtained by DDA and AQUA were compared with values from the literature and a consensus stoichiometry for each of the complexes proposed. AQUA experiment design (right): A549 cells were pulse-stimulated with TNFα and samples collected at 4 time points (time 0 was used as a control). AQUA peptides for TNF-RSC proteins were added to the samples and quantified by targeted proteomics. (**B**) Intensity of the AQUA peptides used in this study falls for about 80% of the peptides within 1 fold $log_{10}$ intensity. **(C)** Linear peptide response at different concentration is evaluated as Pearson correlation. Peptides exhibit linear behavior over up to six orders of magnitude. (**D)** Intensity difference between heavy and light peptides used to quantify the affinity purified TNF-RSC and the proteins present in the lysate. (**E)** Summary of the literature curation, representing possible homooligomeric and heterooligomeric states for each TNF-RSC protein and complex (y axis) across the theoretical or observed molecular weight range (x axis). Selected data about MW as observed by SEC-WB and affinity of protein-protein interactions is also shown.

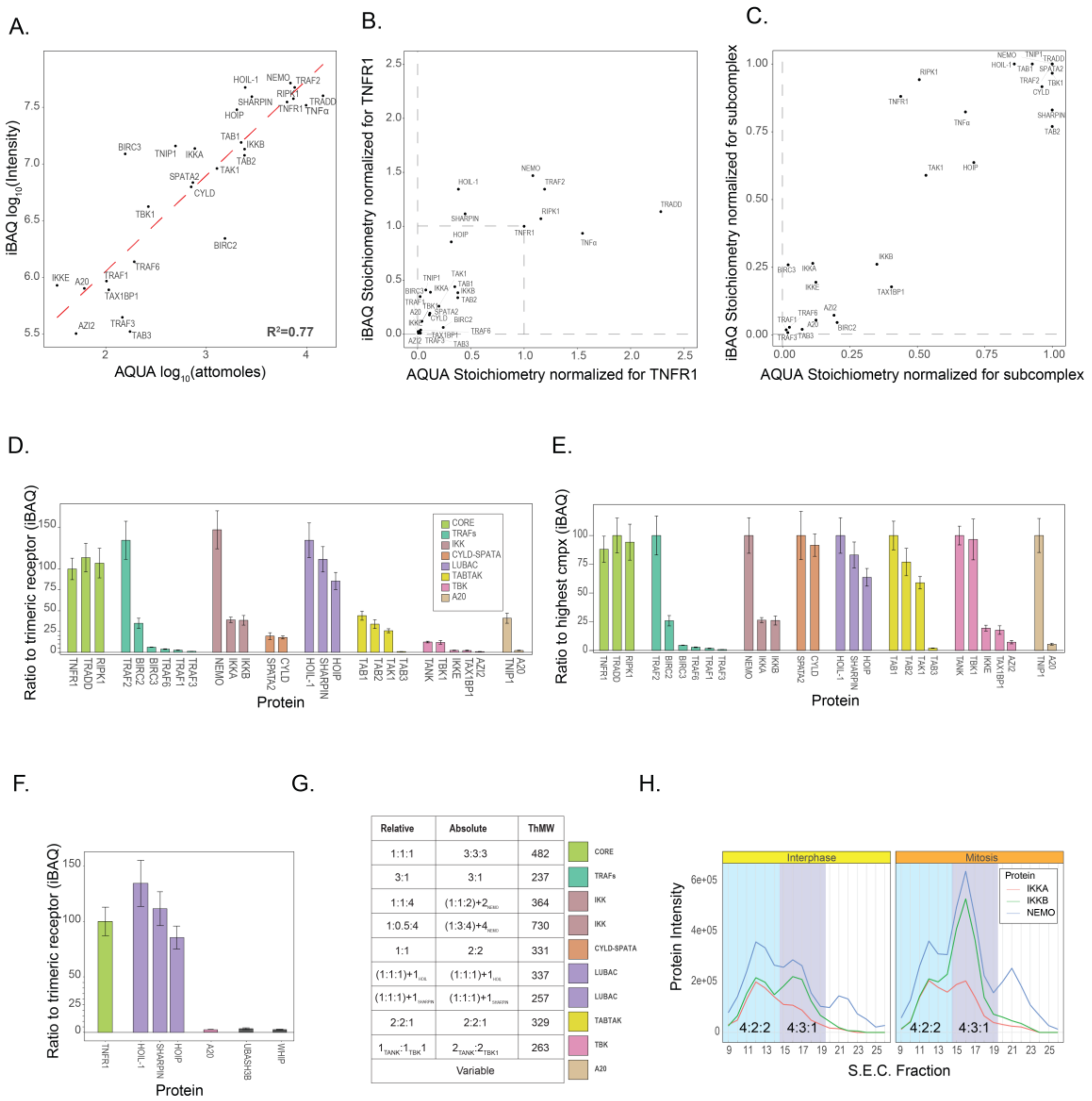


| Relative | Absolute | ThMW | |
|---|---|---|---|
| 1:1:1 | 3:3:3 | 482 | CORE |
| 3:1 | 3:1 | 237 | TRAFs |
| 1:1:4 | $(1:1:2)+2_{NEMO}$ | 364 | IKK |
| 1:0.5:4 | $(1:3:4)+4_{NEMO}$ | 730 | IKK |
| 1:1 | 2:2 | 331 | CYLD-SPATA |
| $(1:1:1)+1_{HOIL}$ | $(1:1:1)+1_{HOIL}$ | 337 | LUBAC |
| $(1:1:1)+1_{SHARPIN}$ | $(1:1:1)+1_{SHARPIN}$ | 257 | LUBAC |
| 2:2:1 | 2:2:1 | 329 | TABTAK |
| $1_{TANK}:1_{TBK}1$ | $2_{TANK}:2_{TBK1}$ | 263 | TBK |
| | Variable | | A20 |

**Figure S12. Assessment of TNF-RSC complexes stoichiometry by orthogonal approaches. (A/B/C)** Correlation between stoichiometries estimated by AP-AQUA-MS and AP-MS (iBAQ) using $log_{10}$ intensities (**A**), stoichiometries normalized to the receptor TNFR1 (**B**) and to the most abundant complex member (**C**). (**D)** iBAQ quantification of TNF-RSC AP-MS DDA data normalized to the trimeric TNFR1 receptor. (**E**) iBAQ quantification of TNF-RSC AP-MS DDA data normalized to most abundant complex member. (**F**) Comparison of UBASH3B and WHIP stoichiometry with LUBAC complex and A20. Data, normalized to the trimeric TNFR1 receptor, are obtained from iBAQ quantification of TNF-RSC AP-MS DDA (**G)** Table reporting suggested relative and absolute stoichiometries for the indicated complexes. These can be considered "consensus" stoichiometries, in that they're derived from the experiments presented in this study combined with evidences from other studies. (**H**) Data from (*19*) indicates the existence of two cytoplasmic isoforms of the IKK complex.

A

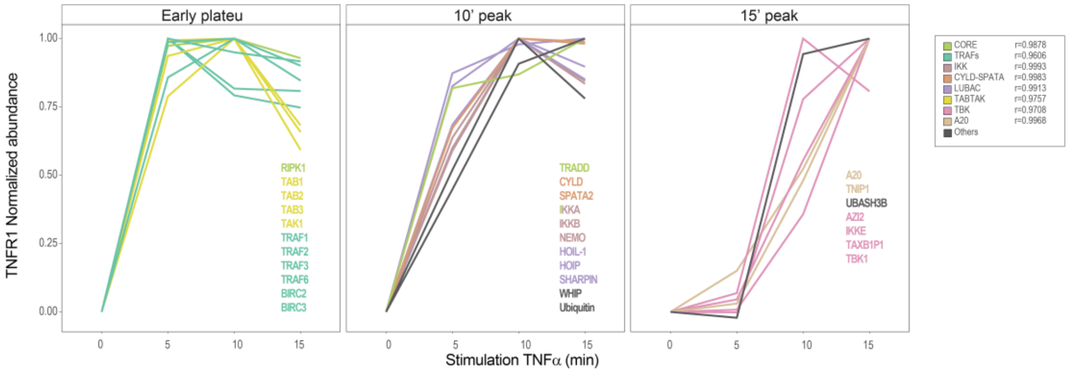


B.

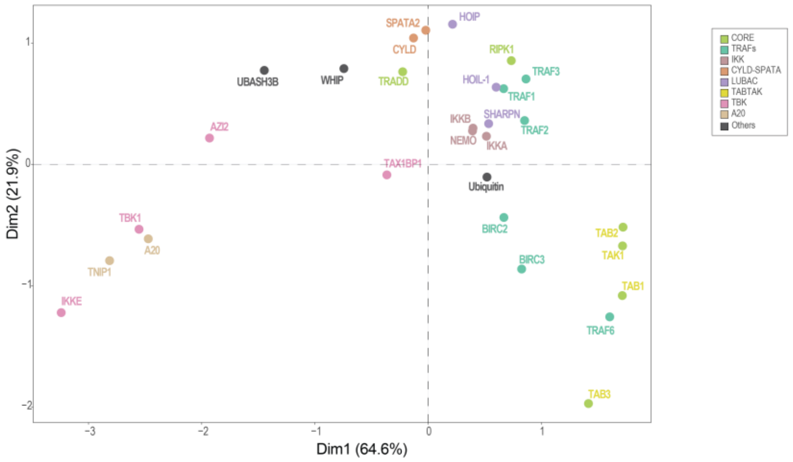


**Figure S13. Time course profiles for the recruitment TNF-RSC members**
**(A)** Time profiles of TNF-RSC proteins at t=0, 5, 10 15 min. Unsupervised hierarchical cluster assigned proteins in three groups (early plateu, 10’ peak and 15’ peak) based on unsupervised hierarchical clustering. Correlation between profiles of proteins belonging to the same complex is shown in the legend. Subsest of this dataset is reported in Figure 2C (UBASH3B, WHIP) and S2D (AZI2, TAX1BP1). **(B)** Principal component analysis of the time profiles shown in panel A.

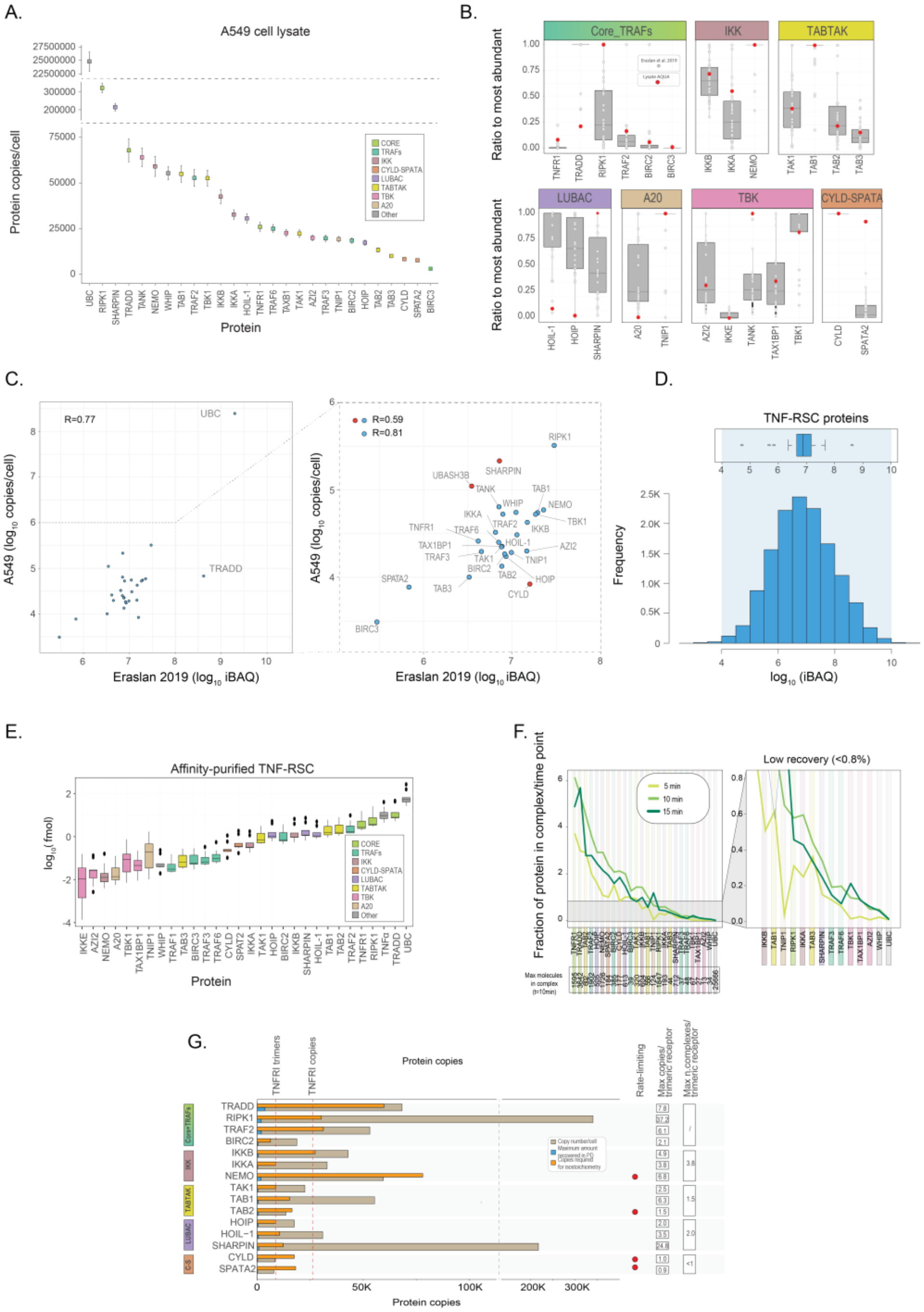


**Figure S14. Abundance and copy/number of TNF-RSC members in lysate and affinity-purified samples. (A)** Distribution of number of copies/cell for the indicated TNF-RSC members as estimated in the lysate-AQUA experiment. (**B**) Relative expression levels of the indicated TNF-RSC proteins as estimated in A549 (red dot) are broadly reflected in average expression levels across 29 human tissues, as determined in (*19*), normalized for the most abundant complex component. (**C**) Expression level correlation between average estimated TNF-RSC members abundance from A549 lysates (this study) and average values from (*20*). **(D)** Abundance distribution of TNF-RSC members based on iBAQ estimate across 29 human tissues (*20*) indicates most proteins have average expression levels. (**E**) Distribution of amount (fmol) of TNF-RSC members estimated in the AP-AQUA-MS experiment (**F**) Calculated fraction of TMF-RSC members recovered (yield of purification) in AP-AQUA-MS experiment across the indicated time points. (**G**) Resource allocation plot. Number of copies/cell (grey bars) are compared with estimated copies isolated from affinity-purified TNF-RSC (blue bars) and the number of copies required to achieve 1:1 stoichiometry with the receptor (orange bars). Data (reported in Dataset 12) are generated from the stoichiometry calculated in AQUA dataset.

**Legends for Dataset 1, 2, 3, 4, 5, 6, 7, 8, 9, 10, 11, 12, 13**

Dataset 1. DDA data (raw files list/mapping; MaxQuant output; statistical evaluation) from AP-MS of the TNF-RSC.
Dataset 2. Known TNF-RSC members and associated identifiers.
Dataset 3. DIA data (raw files list/mapping; input for MapDIA; statistical evaluation) from AP-MS of the TNF-RSC.
Dataset 4. DDA data (raw files list/mapping; MaxQuant output; statistical evaluation) from AP-MS of C- or N-terminally tagged UBASH3B in HEK293 cells. DDA data (raw files list/mapping; MaxQuant output; Saint output) from AP-MS of C- and/or N-terminally tagged LUBAC components
Dataset 5. PRM data (raw files list/mapping; list of selected peptides; Skyline intensities; statistical evaluation) from pulldown of endogenous UBASH3B in A549 cells.
Dataset 6. DEVDase assays data.
Dataset 7. DDA data (raw files list/mapping; maxQuant output; statistical evaluation) from AP-MS of the TNF-RSC in UBASH3B KO A549 cell lines.
Dataset 8. BNPAGE-PRM data (raw files list/mapping; list of selected peptides; Skyline intensities; protein intensities used for plotting).
Dataset 9. Data from AQUA of the isolated TNF-RSC (raw files list/mapping; list of selected peptides; Skyline intensities; protein absolute amounts) and peptides and stoichiometries derived from iBAQ evaluation of DDA data.
Dataset 10. Literature review curating information on size, stoichiometry and binding constants of the TNF-RSC complexes/members from about 100 publications, including (whenever applicable) indications of relevant Fig.s and direct citations.
Dataset 11. Data from absolute quantification and copy number estimation of the TNF-RSC members in A549 lysates (raw files list/mapping; list of selected peptides; Skyline intensities; protein absolute amounts).
Dataset 12. Data used for limiting component calculations, based on AQUA and iBAQ values.
Dataset 13. List of reagents, antibodies, oligonucleotides used in this study.

**SI References**

1. J. Cox, M. Mann, MaxQuant enables high peptide identification rates, individualized p.p.b.-range mass accuracies and proteome-wide protein quantification. *Nat. Biotechnol.* **26**, 1367–1372 (2008).
2. B. MacLean *et al.*, Skyline: an open source document editor for creating and analyzing targeted proteomics experiments. *Bioinformatics*. **26**, 966–968 (2010).
3. Drew, K. *et al.* Integration of over 9,000 mass spectrometry experiments builds a global map of human protein complexes. *Mol. Syst. Biol.* **13**, 932 (2017).
4. Lafont, E. *et al.* TBK1 and IKKε prevent TNF-induced cell death by RIPK1 phosphorylation. *Nat. Cell Biol.* **20**, 1389–1399 (2018).
5. Shembade, N., Harhaj, N. S., Liebl, D. J. & Harhaj, E. W. Essential role for TAX1BP1 in the termination of TNF-α-, IL-1- and LPS-mediated NF-κB and JNK signaling. *Embo J* **26**, 3910–3922 (2007).
6. Hinz, M. & Scheidereit, C. The IκB kinase complex in NF-κB regulation and beyond. *EMBO Rep.* **15**, 46–61 (2014).
7. Pinkas, D. M. *et al.* Structural complexity in the KCTD family of Cullin3-dependent E3 ubiquitin ligases. *Biochem J* **474**, 3747–3761 (2017).
8. Feldman, D. *et al.* Optical Pooled Screens in Human Cells. *Cell* **179**, 787-799.e17 (2019).
9. Mohamed, A., Shah, A. D., Chen, D. & Hill, M. M. RaftProt V2: understanding membrane microdomain function through lipid raft proteomes. *Nucleic Acids Res* **47**, gky948- (2018).
10. Legler, D. F., Micheau, O., Doucey, M.-A., Tschopp, J. & Bron, C. Recruitment of TNF Receptor 1 to Lipid Rafts Is Essential for TNFα-Mediated NF-κB Activation. *Immunity* **18**, 655–664 (2003).
11. Yin, Q. *et al.* E2 interaction and dimerization in the crystal structure of TRAF6. *Nature Structural & Molecular Biology* **16**, 658 (2009).
12. Scholefield, J. *et al.* Super-resolution microscopy reveals a preformed NEMO lattice structure that is collapsed in incontinentia pigmenti. *Nat Commun* **7**, 12629 (2016).
13. Wu, H. & Fuxreiter, M. The Structure and Dynamics of Higher-Order Assemblies: Amyloids, Signalosomes, and Granules. *Cell* **165**, 1055–1066 (2016).
14. Vanamee, É. S. & Faustman, D. L. Structural principles of tumor necrosis factor superfamily signaling. *Sci. Signal.* **11**, eaao4910 (2018).
15. P. D. Mace, C. Smits, D. L. Vaux, J. Silke, C. L. Day, Asymmetric Recruitment of cIAPs by TRAF2. *J Mol Biol* **400**, 8–15 (2010).
16. M. Heusel, *et al.*, Complex-centric proteome profiling by SEC-SWATH-MS. *Mol. Syst. Biol.* **15**, e8438 (2019).
17. M.C. Tanzer *et al.*, Phosphoproteome profiling uncovers a key role for CDKs in TNF signaling. *Nat Commun* **1**, 12 (2021).
18. Li, F. *et al.* Structural insights into the interaction and disease mechanism of neurodegenerative disease-associated optineurin and TBK1 proteins. *Nat Commun* **7**, 1–12 (2016).
19. M. Heusel, *et al.*, A Global Screen for Assembly State Changes of the Mitotic Proteome by SEC-SWATH-MS. *Cell Syst* **10**, 133-155.e6 (2020).
20. Eraslan, B. *et al.* Quantification and discovery of sequence determinants of protein-per-mRNA amount in 29 human tissues. *Mol Syst Biol* 15, e8513 (2019).
