# Supplementary material for: Novel biochemical, structural, and systems insights into inflammatory signaling revealed by contextual interaction proteomics
Source: Proc Natl Acad Sci U S A. 2022 Sep 30;119(40):e2117175119. doi: 10.1073/pnas.2117175119 (PMC9546619; doi:10.1073/pnas.2117175119)
Supplement: Supplementary File [file pnas.2117175119.sd02.pdf]

Name_used_in_this_study	Protein_Complex	Protein	UniprotID	Entry name	Protein names	Gene names (primary)	Gene names (synonym)	Other names	Entrez_Gene	ENSEMBL_ID	Length	Mass
A20	A20	TNFAIP3	P21580	TNAP3_HUMAI	Tumor necrosis f	TNFAIP3	OTUD7C	/	7128	ENSG00000118	790	89,614
TNIP1	A20	TNIP1	Q15025	TNIP1_HUMAN	TNFAIP3-interac	TNIP1	KIAA0113 NAF1	/	10318	ENSG00000145	636	71,864
TNIP2	A20	TNIP2	Q8NFZ5	TNIP2_HUMAN	TNFAIP3-interac	TNIP2	ABIN2 FLIP1	/	79155	ENSG00000168	429	48,700
RIPK1	Core	RIPK1	Q13546	RIPK1_HUMAN	Receptor-interac	RIPK1	RIP RIP1	/	8737	ENSG00000137	671	75,931
TRADD	Core	TRADD	Q15628	TRADD_HUMAI	Tumor necrosis f	TRADD	/	/	8717	ENSG00000102	312	34,247
TNFR1	Core	TNFRSF1A	P19438	TNR1A_HUMAI	Tumor necrosis f	TNFRSF1A	TNFAR TNFR1	/	7132	ENSG00000067	455	50,495
TNFα	Core	TNF	P01375	TNFA_HUMAN	Tumor necrosis f	TNF	TNFA TNFSF2	/	7124,7124,7124,	ENSG00000204	233	25,644
BIRC2	Core/TRAFs	BIRC2	Q13490	BIRC2_HUMAN	Baculoviral IAP r	BIRC2	API1 MIHB RNF4	cIAP1	329	ENSG00000110	618	69,900
BIRC3	Core/TRAFs	BIRC3	Q13489	BIRC3_HUMAN	Baculoviral IAP r	BIRC3	API2 MIHC RNF4	cIAP2	330	ENSG00000023	604	68,372
TRAF1	Core/TRAFs	TRAF1	Q13077	TRAF1_HUMAN	TNF receptor-as	TRAF1	EBI6	/	7185	ENSG00000056	416	46,164
TRAF2	Core/TRAFs	TRAF2	Q12933	TRAF2_HUMAN	TNF receptor-as	TRAF2	TRAP3	/	7186	ENSG00000127	501	55,859
TRAF5	Core/TRAFs	TRAF5	O00463	TRAF5_HUMAN	TNF receptor-as	TRAF5	RNF84	/	7188	ENSG00000082	557	64,406
CYLD	CYLD-SPATA	CYLD	Q9NQC7	CYLD_HUMAN	Ubiquitin carbox	CYLD	CYLD1 KIAA0849	/	1540	ENSG00000083	956	107,316
SPATA2	CYLD-SPATA	SPATA2	Q9UM82	SPAT2_HUMAN	Spermatogenesi	SPATA2	KIAA0757 PD1	/	9825	ENSG00000158	520	58,427
IKKA	IKK	CHUK	O15111	IKKA_HUMAN	Inhibitor of nucle	CHUK	IKKA TCF16	/	1147	ENSG00000213	745	84,640
IKKB	IKK	IKBKB	O14920	IKKB_HUMAN	Inhibitor of nucle	IKBKB	IKKB	/	3551	ENSG00000104	756	86,564
NEMO	IKK	IKBKG	Q9Y6K9	NEMO_HUMAN	NF-kappa-B esse	IKBKG	FIP3 NEMO	/	8517	ENSG00000269	419	48,198
HOIL-1	LUBAC	RBCK1	Q9BYM8	HOIL1_HUMAN	RanBP-type and	RBCK1	C20orf18 RNF54	/	10616	ENSG00000125	510	57,572
HOIP	LUBAC	RNF31	Q96EP0	RNF31_HUMAI	E3 ubiquitin-prot	RNF31	ZIBRA	/	55072,55072	ENSG00000092	1072	119,652
SHARPIN	LUBAC	SHARPIN	Q9H0F6	SHRPN_HUMAI	Sharpin (Shank-a	SHARPIN	SIPL1	/	81858	ENSG00000179	387	39,949
**WHIP**	Other	**WRNIP1**	Q96S55	WRIP1_HUMAI	ATPase WRNIP1	WRNIP1	WHIP	/	56897	ENSG00000124	665	72,133
HTRA2	Other	HTRA2	O43464	HTRA2_HUMAI	Serine protease	HTRA2	OMI PRSS25	/	27429	ENSG00000115	458	48,841
CALCOCO2	Other	CALCOCO2	Q13137	CACO2_HUMAI	Calcium-binding	CALCOCO2	NDP52	/	10241	ENSG00000136	446	52,254
MIB1	Other	MIB1	Q86YT6	MIB1_HUMAN	E3 ubiquitin-prot	MIB1	DIP1 KIAA1323 Z	/	57534	ENSG00000101	1006	110,136
NBR1	Other	NBR1	Q14596	NBR1_HUMAN	Next to BRCA1 g	NBR1	1A13B KIAA0049	/	4077	ENSG00000188	966	107,413
OPTN	Other	OPTN	Q96CV9	OPTN_HUMAN	Optineurin (E3-1	OPTN	FIP2 GLC1E HIP7	/	10133	ENSG00000123	577	65,922
OTULIN	Other	OTULIN	Q96BN8	OTUL_HUMAN	Ubiquitin thioest	OTULIN	FAM105B	/	90268	ENSG00000154	352	40,263
**UBASH3B**	Other	**UBASH3B**	Q8TF42	UBS3B_HUMAI	Ubiquitin-associ	UBASH3B	KIAA1959 STS1	/	84959	ENSG00000154	649	72,696
MIB2	Other	MIB2	Q96AX9	MIB2_HUMAN	E3 ubiquitin-prot	MIB2	SKD ZZANK1	/	142678	ENSG00000197	1013	109,939
TAK1	TABTAK	MAP3K7	O43318	M3K7_HUMAN	Mitogen-activate	MAP3K7	TAK1	/	6885	ENSG00000135	606	67,196
TAB1	TABTAK	TAB1	Q15750	TAB1_HUMAN	TGF-beta-activat	TAB1	MAP3K7IP1	/	10454	ENSG00000100	504	54,644
TAB2	TABTAK	TAB2	Q9NYJ8	TAB2_HUMAN	TGF-beta-activat	TAB2	KIAA0733 MAP3I	/	23118	ENSG00000055	693	76,494
TAB3	TABTAK	TAB3	Q8N5C8	TAB3_HUMAN	TGF-beta-activat	TAB3	MAP3K7IP3	/	257397	ENSG00000157	712	78,653
AZI2	TBK	AZI2	Q9H6S1	AZI2_HUMAN	5-azacytidine-inc	AZI2	NAP1 TBKBP2	/	64343	ENSG00000163	392	44,935
IKKE	TBK	IKBKE	Q14164	IKKE_HUMAN	Inhibitor of nucle	IKBKE	IKKE IKKI KIAA01	/	9641	ENSG00000263	716	80,462
TANK	TBK	TANK	Q92844	TANK_HUMAN	TRAF family mer	TANK	ITRAF TRAF2	/	10010	ENSG00000136	425	47,816
TAX1BP1	TBK	TAX1BP1	Q86VP1	TAXB1_HUMAN	Tax1-binding prc	TAX1BP1	T6BP	/	8887	ENSG00000106	789	90,877
TBK1	TBK	TBK1	Q9UHD2	TBK1_HUMAN	Serine/threonine	TBK1	NAK	/	29110	ENSG00000183	729	83,642
TRAF3	TRAFs	TRAF3	Q13114	TRAF3_HUMAN	TNF receptor-as	TRAF3	CAP1 CRAF1	/	7187	ENSG00000131	568	64,490
TRAF6	TRAFs	TRAF6	Q9Y4K3	TRAF6_HUMAN	TNF receptor-as	TRAF6	RNF85	/	7189	ENSG00000175	522	59,573
Ubiquitin	Ubiquitin	UBC	P0CG48	UBC_HUMAN	Polyubiquitin-C [	UBC		/	7316	ENSG00000150	685	77,039
Ubiquitin	Ubiquitin	RPS27A	P62979	RS27A_HUMAN	Ubiquitin-40S rib	RPS27A	UBA80 UBCEP1	/	6233	ENSG00000143	156	17,965
Ubiquitin	Ubiquitin	UBA52	P62987	RL40_HUMAN	Ubiquitin-60S rib	UBA52	UBCEP2	/	7311	ENSG00000221	128	14,728
Ubiquitin	Ubiquitin	UBB	P0CG47	UBB_HUMAN	Polyubiquitin-B [	UBB		/	7314	ENSG00000170	229	25,762
