# Supplementary material for: Novel biochemical, structural, and systems insights into inflammatory signaling revealed by contextual interaction proteomics
Source: Proc Natl Acad Sci U S A. 2022 Sep 30;119(40):e2117175119. doi: 10.1073/pnas.2117175119 (PMC9546619; doi:10.1073/pnas.2117175119)
Supplement: Supplementary File [file pnas.2117175119.sd13.pdf]

REAGENT or RESOURCE	SOURCE	IDENTIFIER
**Antibodies**		
Actin	Abcam	ab179467
Alpha-Tubulin	Thermo Fisher Scientific	A11126
p38	Santa Cruz	sc-7972
P-p38 (T180/Y182)	Cell Signaling	9215S
ikBa	Santa Cruz	sc-203
P- ikBa (S32/36)	Cell Signaling	9246
p65	Cell Signaling	8242S
P- p65 (S536)	Cell Signaling	3033S
ERK 1/2	Cell Signaling	4695S
P-ERK 1/2 (T202/Y204)	Cell Signaling	4370S
JNK2	Cell Signaling	9258S
P-JNK (T183/T185)	BioConcept	98F2
UBASH3B	Santa Cruz	sc-514612
UBASH3B	Eurogentec, in-house designed	/
WHIP	Santa Cruz	sc-377402
WHIP	Eurogentec, in-house designed	/
RIPK1	BDBiosciences	610459
Mouse (Secondary)	Jackson Immuno Research Labs	115035003
Rabbit (Secondary)	Cell Signaling	7074S
GADPH	EMD Millipore	MAB374
**Chemicals, Peptides, and Recombinant Proteins**		
Trypsin-EDTA	Life Technologies Europe BV	25300-054
X-tremeGENE 9 DNA Transfection Reagent	Sigma Aldrich Chemie	6365787001
Blasticidin S – Hydrochlorid	HUBERLAB.AG	A3784.0025
B-PER™ Bacterial Protein Extraction Reagent	Thermo Fisher Scientific	78243
cOmplete™, EDTA-free Protease Inhibitor Cocktail	Roche	11873580001
DNAse I	Roche	10104159001
PreScission Protease	GE Healthcare	27-0843-01
USP21 Catalytic Domain, GST tag	Boston Biochem	E-622
PMSF	Sigma-Aldrich	P7626-5G
Avidin	IBA Life Sciences	2-0204-015
TCEP	Sigma-Aldrich	C4706-2G
IAA	Sigma-Aldrich	I6125-5G
Sequencing grade modified trypsin (100µg)	Promega	V5113
Lysyl Endopeptidase	Wako	WA3 125-05061
NuPAGE™ MOPS SDS Running Buffer (20X)	Thermo Fisher Scientific	NP0001
NuPAGE™ MES SDS Running Buffer (20X)	Thermo Fisher Scientific	NP0002
Lipofectamine RNAiMAX	Thermo Fisher Scientific	13778-150

Lipofectamine RNAiMAX	Thermo Fisher Scientific	13778-150
IGEPAL	Sigma-Aldrich Chemie GmbH	8896
Methanol	Fisher Chemicals	M/4058/17-4
Acetonitrile	Fisher Chemicals	A995-212-4
Formic Acid, LC-MS grade	Pierce	28905
3X Flag Peptide	Sigma-Aldrich Chemie GmbH	F4799
Glycerol	Chemie Brunschwig AG	15892-0010
Ethanol	Honeywell	2860
Hydrochloric Acid	VWR International GmbH	1.00317.1000
Acetic Acid	VWR International GmbH	1.00063.1000
Hepes	Sigma Aldrich Chemie GmbH	H4034
DTT	Sigma Aldrich Chemie GmbH	D0632
Sodium chloride	Merck	1.06404.5000
EDTA	Biosolve	5142391
Penicillin-Streptomycin	Sigma Aldrich Chemie GmbH	P0781-100ML
Foetal Calf serum (FCS) Mycoplasma and Virus screened 500ml	BioConcept AG	2-01F10-I
Hygromycin B liquid (50 mg/ml) 20ml	Invitrogen	10687010
**Critical Commercial Assays**		
QIAprep Spin Miniprep Kit	QIAGEN	27104
ECL™ Prime Western Blotting System	GE Healthcare	RPN2232
Pierce™ Quantitative Colorimetric Peptide Assay	Thermo Fisher Scientific	23275
Pierce™ BCA Protein Assay Kit	Thermo Fisher Scientific	23225
**Deposited Data**		
SEC-MS data	Heusel et al., 2020	https://sec-explorer.shinyapps.io/hela_cellcycle/
Bioplex v.3	Gygi laboratory, Harvard University	https://bioplex.hms.harvard.edu/
Biogrid	Oughtred et al., 2019	https://thebiogrid.org/
CORUM	Giurgiu et al., 2019	http://mips.helmholtz-muenchen.de/corum/
Raw Mass Spectrometry data	This study	ProteomeXChange (final identifiers to be updated)
Skyline/Quantification/Parameter files	This study	ProteomeXChange (final identifiers to be updated)
Proteome Profiling of 29 human tissues	Eraslan et al., 2019	https://www.proteomicsdb.org/
**Experimental Models: Cell Lines**		
A549 cells	ATCC	ATCC® CCL-185™
Flp-Ln T-Rex 293 Cell Line	Thermo Fisher Scientific (Invitrogen)	R78007
**Oligonucleotides**		
Refer to table 7	N/A	N/A
**Recombinant DNA**		
pTOSH-GW-FRT-HA-Strep	Glatter et al., 2009	N/A
pOG44 Flp recombinase expression vector	Invitrogen	V600520
hORFeome V5.1	Horizon Discovery/ Dharmacon	Open Biosystem
**Software and Algorithms**		
MaxQuant	Cox and Mann, 2008	https://www.maxquant.org/ RRID:SCR_014485
Spectronaut	Biognosys	https://biognosys.com/
Skyline	Maclean et al., 2010	https://skyline.ms/project/home/begin.view? RRID:SCR_014080
DAVID Bioinformatics Resources 6.8	DAVID, NCI	https://david-d.ncifcrf.gov/ RRID:SCR_001881
ImageJ/FiJi	Schindelin et al., 2012	https://imagej.net/Welcome

ImageJ/Fiji	Schindelin et al., 2012	RRID:SCR_003070
R	The R Project	https://www.R-project.org RRID:SCR_000432
Cytoscape 3.6.0	Kohl et al, 2011	http://www.cytoscape.org RRID:SCR_003032
**Other**		
Amersham Protran 0.2 NC supported Western blotting membranes	GE Healthcare	10600015
Amersham Hybond P 0.45 PVDF blotting membrane	GE Healthcare	10600023
UltraMicroSpin Columns, 2-100µl (3-30µg), 96/pk	The Nest group	SUM SS18V
Self-Pack Picofrit columns 75um ID, 10 um tip, no coating	Picofrit	PF360-75-10-N
EASY-Spray™ HPLC Columns	Thermo Scientific™	ES806A
Glutathione Sepharose 4 Fast Flow-25ml	Sigma Aldrich Chemie GmbH	GE17-5132-01
Superdex 75 10/300	GE Healthcare	GE17-5174-01
ANTI-FLAG M2 AFFINITY GEL	Sigma-Aldrich Chemie GmbH	A2220-5M
**SiRNA**		
TARGET/NAME	MANUFACTURER	CAT #
AllStars Neg. siRNA AF 488 (5 nmol)	QIAGEN	1027284
UBASH3B	QIAGEN	Hs_STS-1_1 FlexiTube siRNA (SI00144837)
UBASH3B	QIAGEN	Hs_STS-1_5 FlexiTube siRNA (SI02647022)
UBASH3B	QIAGEN	Hs_STS-1_7 FlexiTube siRNA (SI02647036)
UBASH3B	QIAGEN	Hs_STS-1_8 FlexiTube siRNA (SI03091816)
WHIP	QIAGEN	Hs_WRNIP1_5 FlexiTube siRNA (SI03019492)
WHIP	QIAGEN	Hs_WRNIP1_7 FlexiTube siRNA (SI04158602)
WHIP	QIAGEN	Hs_WRNIP1_8 FlexiTube siRNA (SI04175430)
WHIP	QIAGEN	Hs_WRNIP1_9 FlexiTube siRNA (SI04181779)
WHIP	QIAGEN	Hs_WRNIP1_10 FlexiTube siRNA (SI04223380)

**OLIGONUCLEOTIDES**	
NAME	SEQUENCE
UBASH3b_gRNA_1_exon3_forward	5-CACCGAGGTAGAGGACGTACTCCCG-3
UBASH3b_gRNA_1_exon3_reverse	5-AAACCGGGAGTACGTCCTCTACCTC-3
UBASH3b_gRNA_2_exon4_forward	5-CACCGTGGACGACGTATAGAGCTCC-3
UBASH3b_gRNA_2_exon4_reverse	5-AAACGGAGCTCTATACGTCGTCCAC-3
WRNIP1_gRNA_1_exon1_forward	5-CACCGTGCGAGTTGATGTGCGCGGC-3
WRNIP1_gRNA_1_exon1_reverse	5-AAACGCCGCGCACATCAACTCGCAC-3
WRNIP1_gRNA_2_exon1_forward	5-CACCGCGTGTCAGGACGCATCGTGT-3
WRNIP1_gRNA_2_exon1_reverse	5-AAACACACGATGCGTCCTGACACGC-3

**gRNA TARGET SEQUENCES**	
NAME	SEQUENCE
gRNA_1_UBASH3b_exon3	AGGTAGAGGACGTACTCCCG
gRNA_2_UBASH2b_exon4	TGGACGACGTATAGAGCTCC
gRNA_1_WRNIP1_exon1	TGCGAGTTGATGTGCGCGGC
gRNA_2_WRNIP1_exon1	CGTGTCAGGACGCATCGTGT
